# A scientometric review of blue hydrogen production

**DOI:** 10.1007/s43937-026-00203-x

**Published:** 2026-10-17

**Authors:** Monasib Romel, Golam Kabir, Bashirul Haq

**Affiliations:** 1 Industrial Systems Engineering, University of Regina, 3737 Wascana Pkwy, Regina, SK S4S 0A2 Canada; 2 Department of Industrial and Systems Engineering, King Fahd University of Petroleum & Minerals, Dhahran, Saudi Arabia; 3 Department of Petroleum Engineering, The College of Petroleum, King Fahd University of Petroleum & Minerals, Engineering & Geosciences, Dhahran, Saudi Arabia

**Keywords:** Blue hydrogen production, Carbon capture and storage (CCS), Bibliometric analysis, Decarbonization, Life cycle assessment (LCA)

## Abstract

Blue hydrogen, produced from natural gas with carbon capture and storage (CCS) is increasingly viewed as a key pathway for large-scale hydrogen supply and energy system decarbonization. This study analyzes 1432 publications indexed in the Web of Science Core Collection (WOSCC) (2000–2025) to examine the global evolution of blue hydrogen research. Bibliometric methods were applied using Bibliometrix for performance analysis, VOSviewer for collaboration and keyword mapping, and CiteSpace for cluster and trend analysis. Results show limited activity before 2010, followed by rapid growth after 2018 and strong acceleration in the past five years. China, the United States, and Europe lead in output, with growing contributions from the Middle East and South Asia. Collaboration networks indicate increasing international partnerships. Major research themes include steam methane reforming (SMR) with CCS, techno-economic and life-cycle assessments, capture and purification technologies, transport and storage logistics, and policy frameworks. Emerging topics include supply chain decarbonization, methane leakage, infrastructure planning, and carbon credit mechanisms. The findings provide a comprehensive map of trends, gaps, and future research directions in blue hydrogen.

**Supplementary Information:**

The online version contains supplementary material available at https://doi.org/10.1007/s43937-026-00203-x.

## Introduction

According to the International Energy Agency’s Global Hydrogen Review series, global hydrogen production increased from 90 million tons in 2020 to 94 million tons in 2021 and 95 million tons in 2022 [1–5]. Production subsequently reached 97 million tons in 2023, 100 million tons in 2024, and more than 100 million tons in 2025 [1–3]. This growth is and in line with overall global energy demand growth. Almost two-thirds of hydrogen is produced from natural gas reforming and coal-based production remains the second-largest share [2]. Low-emission hydrogen contributes less than 1% of total supply which indicates limited penetration of clean technologies [1]. The supply of hydrogen is dominated by fossil fuels where 290 billion cubic meters natural gas and 90 million tons of coal have been used in the year of 2024 [1]. This production is largely dominated by fossil fuels with 62% from natural gas, 19% from coal, and 18% as by-product hydrogen and oil contributed less than 1% [3]. Low-emissions hydrogen production grew by 10% in 2024 and is on track to reach 1 million ton in 2025 but it still accounts for less than 1% of global production [1]. The reliance on fossil-based hydrogen resulted in approximately 980 million tons of CO₂ emissions in 2024 which is about 3% higher than 2023 [1]. Approximately 15 fossil fuel-based hydrogen facilities are currently equipped with CCS. Together, they capture about 12 million tons of CO₂ annually while producing 0.9–1.2 million tons of hydrogen per year [2]. Natural gas reforming facilities captured approximately 4 million tons of CO₂ and produce 0.35 million tons of hydrogen [2]. Coal and oil gasification facilities captured approximately 8 million tons of CO₂ and produce 0.3 million tons of hydrogen. Hydrogen-related projects now represent more than 25% of the global CO₂ capture capacity under development [2]. Low-emission hydrogen production using CCS is projected to increase from 0.6 million tons in 2023 to approximately 11 million tons annually by 2030 [2].

The low-emission hydrogen sector faces high production costs, policy uncertainty, slow infrastructure development, and project delays or cancellations [1]. More than 200 projects have been announced since 2020. Low-emission hydrogen production is expected to increase from less than 1% of global production today to approximately 4% by 2030 [1]. The production will reach approximately 4.2 million tons per year with an additional 6 million tons having deployment potential under supportive policy conditions [1]. Almost all hydrogen is still used in traditional sectors and produced from fossil fuels which is leading to high CO₂ emissions [4]. Adjustment between supply and demand gap will be key for low-emission hydrogen to mitigate the 2030 net-zero climate targets [4]. Under the net-zero pathway, hydrogen production through steam methane reforming with CCS is projected to reach 10–15 million tons in 2030. Production could increase to 150–170 million tons by 2040 and approximately 200 million tons by 2050 [6].

Integrating hydrogen into modern energy systems requires coordinated government intervention and policy support [7]. The IEA identifies five priorities for accelerating deployment: establishing long-term targets, creating demand, reducing investment risks, supporting research and innovation, and harmonizing standards [7]. Hydrogen policies, investments, and projects are expanding. Current deployment remains well below the levels required to meet the 2030 and 2050 targets. Renewable hydrogen currently costs approximately USD 3.00 to 8.00 per kg, but this range is projected to decline to USD 1.00 to USD 3.50 per kg by 2030 [5].

Hydrogen is classified by color to indicate its production pathway and carbon intensity. Green hydrogen is produced via renewable-powered electrolysis with near-zero emissions [8]. Blue hydrogen is generated from natural gas using SMR or ATR with CCS which can reduce CO₂ emissions by up to 90% [1]. A large share of global hydrogen is generated from natural gas steam reforming which emits significant CO₂ which makes the conventional fossil-based hydrogen environmentally unsustainable [9].

Conventional hydrogen production begins with natural-gas desulfurization, followed by SMR and water–gas shift reactions. At high temperatures, methane reacts with steam to produce hydrogen and CO₂, as shown in Eqs. (1)–(3) [13, 14].

1 $$2{\mathrm{H}}_{2}\mathrm{S}+ {\mathrm{SO}}_{2} \to 3\mathrm{S}+3{\mathrm{H}}_{2}\mathrm{O}$$

2 $${\mathrm{CH}}_{4}+ {\mathrm{H}}_{2}\mathrm{O} \to \mathrm{C}\mathrm{O}+3{\mathrm{H}}_{2}$$

3 $$\mathrm{C}\mathrm{O}+ {\mathrm{H}}_{2}\mathrm{O} \to {\mathrm{CO}}_{2}+ {\mathrm{H}}_{2}$$

SMR remains the dominant blue hydrogen technology which can achieve up to 90% CO₂ capture with about 60% of emissions available from concentrated streams for efficient capture [12, 15]. ATR can capture from 95 to 99% of CO₂, while partially oxidized method and coal gasification enable from 90 to 95% capture rates [4]. On the other hand, biomass gasification with CCS offers carbon removal potential with California’s first commercial biomass-to-hydrogen plant expected to capture 0.15 million tons CO₂ annually from 2025 [16]. Higher CO₂ capture is a growing research focus to move toward near-carbon–neutral production [29]. The low volumetric energy density of hydrogen presents storage and transport challenges which require innovations such as compression, chemical carriers, and underground storage for large-scale and seasonal applications [17, 18]. Effective policy and regulatory frameworks are crucial for deployment along with financial incentives such as the U.S. 45Q tax credit to support CCS-Integrated hydrogen projects [19, 20]. On the other hand, hybrid model combining blue and green hydrogen is expected to dominate the early hydrogen economy which can leverage existing infrastructure while green hydrogen technologies [21, 22].

This study systematically reviews existing research on blue hydrogen production to analyze emerging trends. Using the Preferred Reporting Items for Systematic Reviews and Meta-Analyses (PRISMA) framework, it collects, evaluates, and synthesizes scholarly literature into thematic sustainability categories and identifies future research directions. Existing studies on blue hydrogen have some research gap. Much of the literature examines individual dimensions such as production technology, techno-economic performance, environmental impacts, carbon capture efficiency, or policy separately. The interactions among technological development, environmental performance, economic feasibility, infrastructure requirements, and policy support remain insufficiently integrated. Previous studies provide limited longitudinal and network-based evidence on how blue hydrogen research has evolved across time, disciplines, countries, institutions, and research communities. In particular, publication performance, collaboration structures, subject-category evolution, thematic relationships, and emerging research frontiers have rarely been examined within a unified analytical framework. The relationship between academic research leadership and practical industrial deployment remains insufficiently explored. This paper addresses these deficiencies through three major contributions.

1. A comprehensive global assessment of research performance and collaboration.
2. An integrated explanation of the intellectual and thematic evolution of blue hydrogen research.
3. A research-to-deployment synthesis that converts bibliometric evidence into an actionable research agenda.

## Methodology

**Fig. 1:**
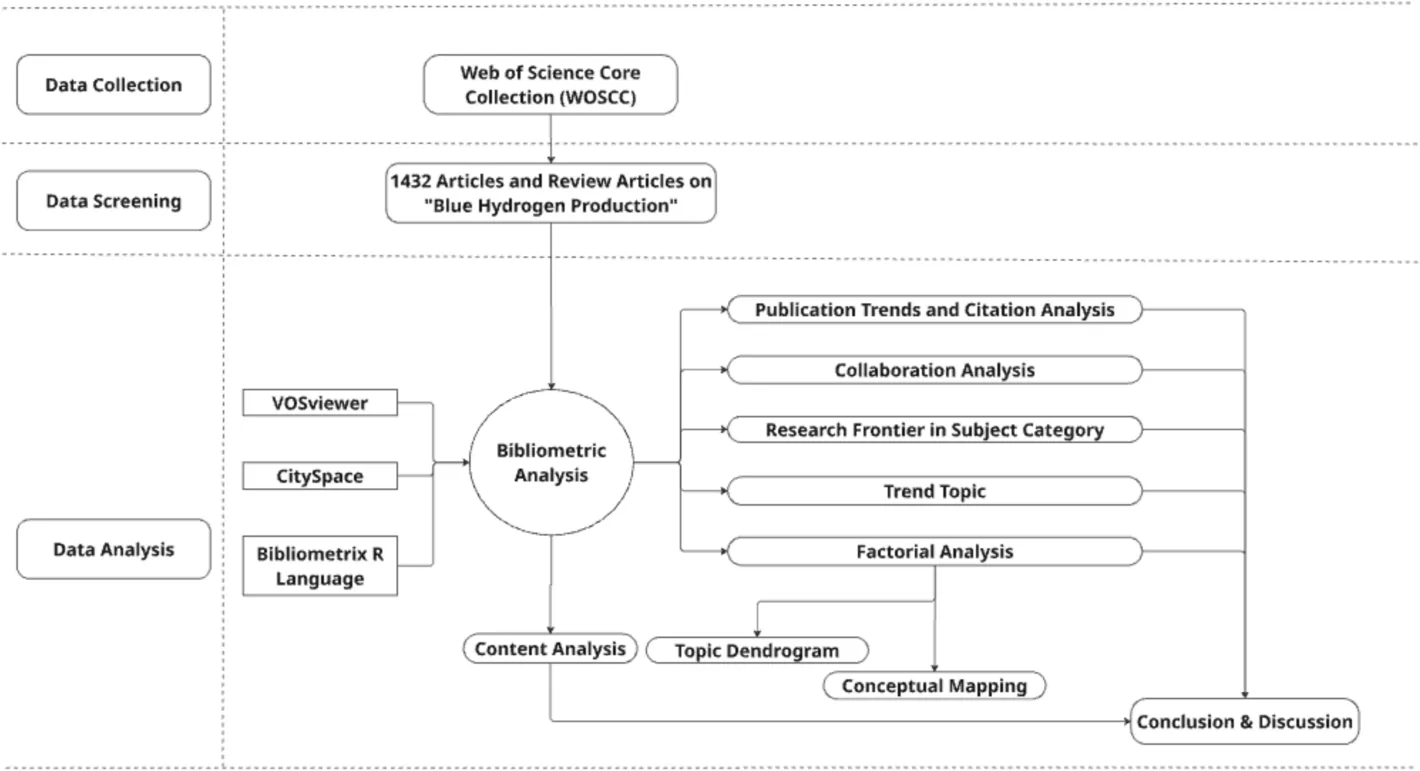
Bibliometric analysis framework

Figure 1 describes the data investigation method for blue hydrogen production research. The methodology is divided into three steps data collection, data screening and data analysis.

### Data Collection

The WOSCC has been used in this study as data source for bibliometric analysis of blue hydrogen production from the years 2000 to 2025. The WOSCC provides comprehensive database for bibliometric analysis across a wide array of disciplines including the natural sciences, social sciences, and humanities [23, 24]. It accommodates bibliometric studies by offering essential analytical tools and data for researchers aiming to assess the impact of publications and identify emerging themes within their fields [25, 26]. The bibliometric techniques that leverage WOSCC include citation analysis, co-citation analysis, and keyword co-occurrence mapping. These methods facilitate the visualization of knowledge networks and allow researchers to ascertain influential publications and authors [27]. This time frame has helped this study to identify the shifts in research focus and the evolution of specific fields.

### Data screening

A systematic screening process was applied to ensure data relevance and quality. The search was conducted using the keyword phrase Blue Hydrogen Production in the Abstract field in only English Language publications. The analysis covered publications from 2000 to 2025. This period captures both the early conceptual development of blue hydrogen research and its recent technological advances. An initial search yielded 1491 publications. Filters were applied to include only “Article” and “Review Article” categories to refine the dataset and focus on scholarly contributions. Other publication types such as conference proceedings, book chapters, editorial materials and notes were excluded as they often lack complete methodological and analytical content necessary for bibliometric evaluation. The unrelated publications of our study were removed from the dataset. After this refinement, a total of 1432 publications were retained for analysis. The retrieved records included comprehensive bibliographic information such as titles, author names, affiliations, publication years, source journals, abstracts, author keywords, and cited references. These data were exported in plain text (.txt) format with the “Full Record and Cited References” to facilitate subsequent processing and analysis in VOSviewer, CiteSpace and Bibliometrix R Language. Duplicate entries and incomplete records were manually checked and removed to ensure data integrity. This cleaned dataset of 1432 publications formed the foundation for subsequent analysis, keyword co-occurrence mapping and collaboration network visualization.

### Data analysis

The retrieved dataset was analyzed using VOSviewer (1.6.20), CiteSpace (6.1.R2 Advanced), and the Bibliometrix package in R to study quantitative evaluation of research works. [28–30]. Researchers leverage the integration of WOSCC data with software tools such as CiteSpace and VOSviewer to enhance their bibliometric analyses and visualization [31, 32]. The bibliometric method integrates mathematical, statistical, and linguistic principles. This study emphasizes the quantitative evaluation of research output and content-related indicators. It is primarily applied to explore the development patterns, interconnections, and interdisciplinary influences within a specific research domain and thereby identifying potential emerging growth areas. The use of visualization tools such as charts and tables helps to describe the research hotspots and supports quantitative analysis of the field of blue hydrogen production [33–35].

VOSviewer is used to construct and visualize co-authorship, keyword co-occurrence, and institutional collaboration networks [36]. CiteSpace 6.1.R2 Advanced was used to construct and visualize knowledge networks. Cluster quality was evaluated using modularity and silhouette values. These metrics supported the identification of the field’s intellectual structure and evolution [37–39]. Bibliometrix (R package) is used for bibliometric indicators, build collaboration, co-word networks, and generate mapping visualizations like trends, dendrograms, conceptual maps for quantitative literature analysis [40–43].

## Results

The temporal evolution of blue hydrogen research was divided into three developmental stages. The foundational and exploratory phase (2000–2010) was characterized by limited publication activity and chemistry-oriented studies. The technological expansion and CCS-integration phase (2010–2020) introduced greater attention to catalytic processes, environmental engineering, and carbon capture. The interdisciplinary and deployment-oriented phase (2020–2025) focused on sustainability, techno-economic analysis, renewable integration, policy, and industrial deployment. To explain the obtained results, the temporal evolution of blue hydrogen research was interpreted using three broader developmental stages.

i. Foundational and exploratory research phase from 2000 to 2010 which is characterized by limited publication activity and chemistry-oriented studies;
ii. Technological expansion and CCS integration phase from 2010 to 2020 which is characterized by increasing attention toward catalytic processes, environmental engineering, and carbon capture integration; and
iii. Interdisciplinary and deployment-oriented phase from 2020 to 2025 which is characterized by rapid publication growth, sustainability assessment, techno-economic analysis, renewable integration, policy studies, and industrial deployment.

**Fig. 2:**
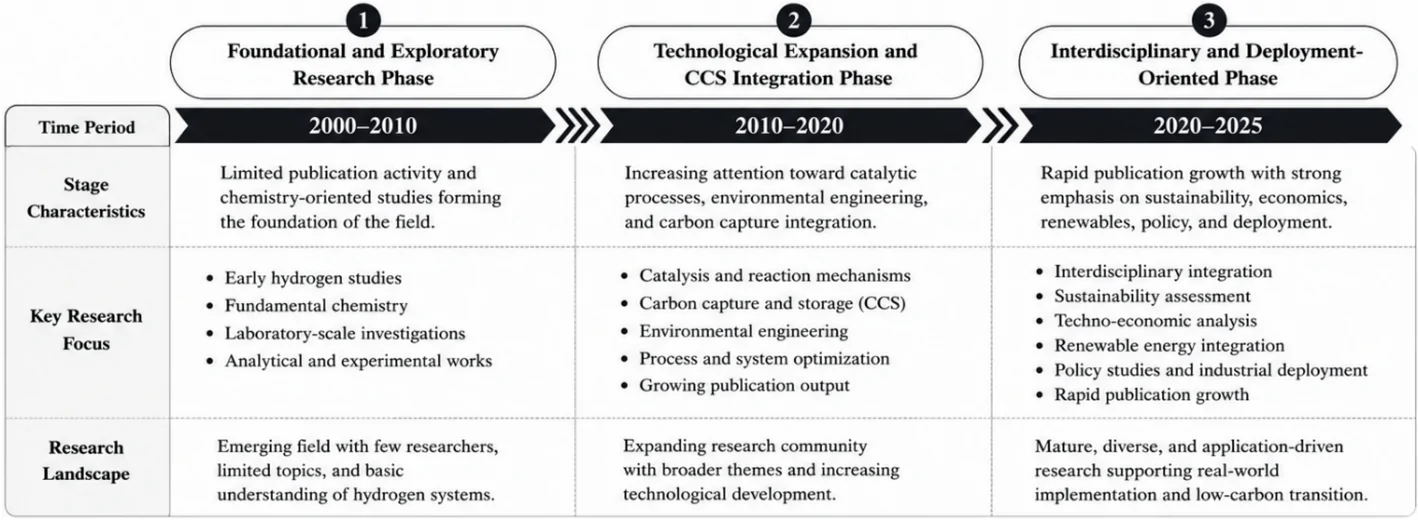
Temporal Evolution of Blue Hydrogen Research

Minor variations in transition from differences in analytical methods including publication trend analysis, CiteSpace timeline clustering, and Bibliometrix topic evolution analysis. However, all analyses consistently indicate a major shift toward integrated low-carbon hydrogen systems from Technological expansion and CCS integration phase. Figure 2 illustrates the temporal evolution of blue hydrogen research from foundational chemistry-based studies to CCS-integrated technological development and finally to interdisciplinary, sustainability-oriented, and deployment-focused research.

### Global research context and publication trends

**Fig. 3:**
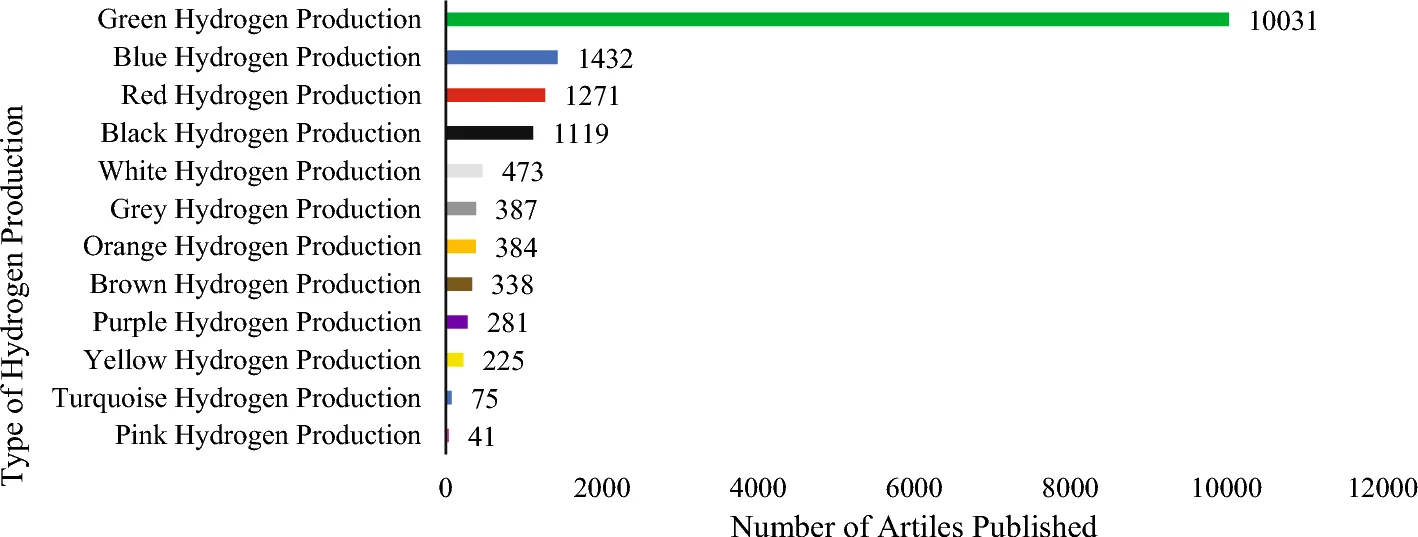
Global research distribution of hydrogen production pathways (2000–2025)

A total of 1432 publications related to Blue Hydrogen Production were retrieved from the WOSCC for the period 2000 to 2025 for Research & Review Articles category. These studies have collectively received 42,065 citations, averaging 29.38 citations per article, with an h-index of 92, underscoring the substantial impact and relevance of this research field. Figure 3 shows the number of articles and reviews published between 2000 and 2025. Green hydrogen production is the main focus of the research interest with over 10,000 publications. Blue hydrogen ranks second with 1432 publications with sustained focus on low-carbon hydrogen from natural gas with CCS integration. Emerging pathways such as red (1271), black (1119), and purple (281) hydrogen show moderate research visibility. Turquoise and pink hydrogen remain in early exploratory stages together accounting for fewer than 120 publications.

**Fig. 4:**
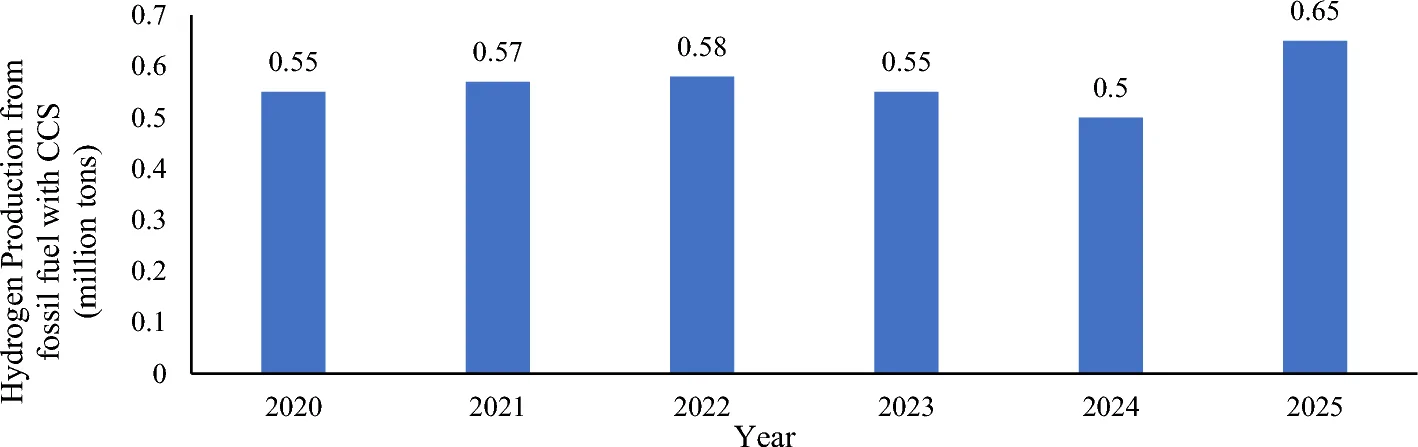
Blue hydrogen production from 2020 to 2025 [1]

Figure 4 illustrates the growth of blue hydrogen production from 2020 to 2025. Global blue hydrogen capacity is projected to reach 5 million tons per year by 2030 driven by CCS-Integrated projects in North American region [10]. About half of this capacity is being developed within industrial clusters connected to CO₂ transport and storage networks [4]. Almost 80% of these projects are in Europe and 15% in Canada which indicates strong regional momentum for large-scale deployment [4]. Japan and Korea are expected to create annual demand for 1–1.5 million tons of hydrogen-equivalent low-carbon ammonia by 2030. This demand will be driven by ammonia co-firing policies and dependence on imports resulting from limited domestic energy resources [11, 12].

**Fig. 5:**
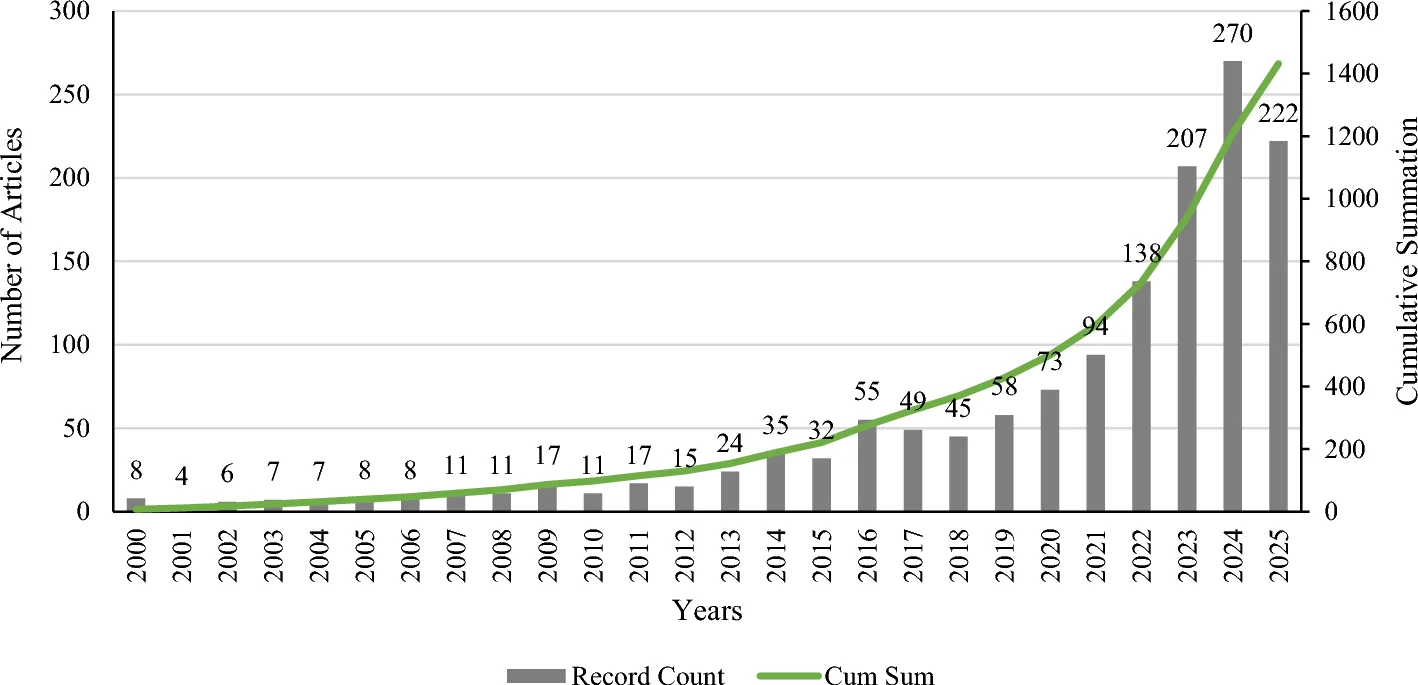
Annual and cumulative number of publications on Blue Hydrogen Production

Figure 5 shows the publication pattern on Blue Hydrogen Production which has steady evolution over the last twenty-five years. During the foundational research phase, the number of publications was low and topics were still emerging and had limited attention. During the technological expansion phase, the output started to rise more clearly and started moving from small numbers to around fifty papers per year. The annual publications grew steadily into the range of 60 to 90 which reflects wider adoption of the topic across different research groups. During the interdisciplinary and deployment-oriented phase publications reached the peak of about 270 publications. Blue Hydrogen Production had become a major focus area for the last five years or so. The cumulative growth curve continues to rise rapidly and surpassed 1400 total publications by 2024 and showing the strong momentum of the field in the upcoming years.

**Fig. 6:**
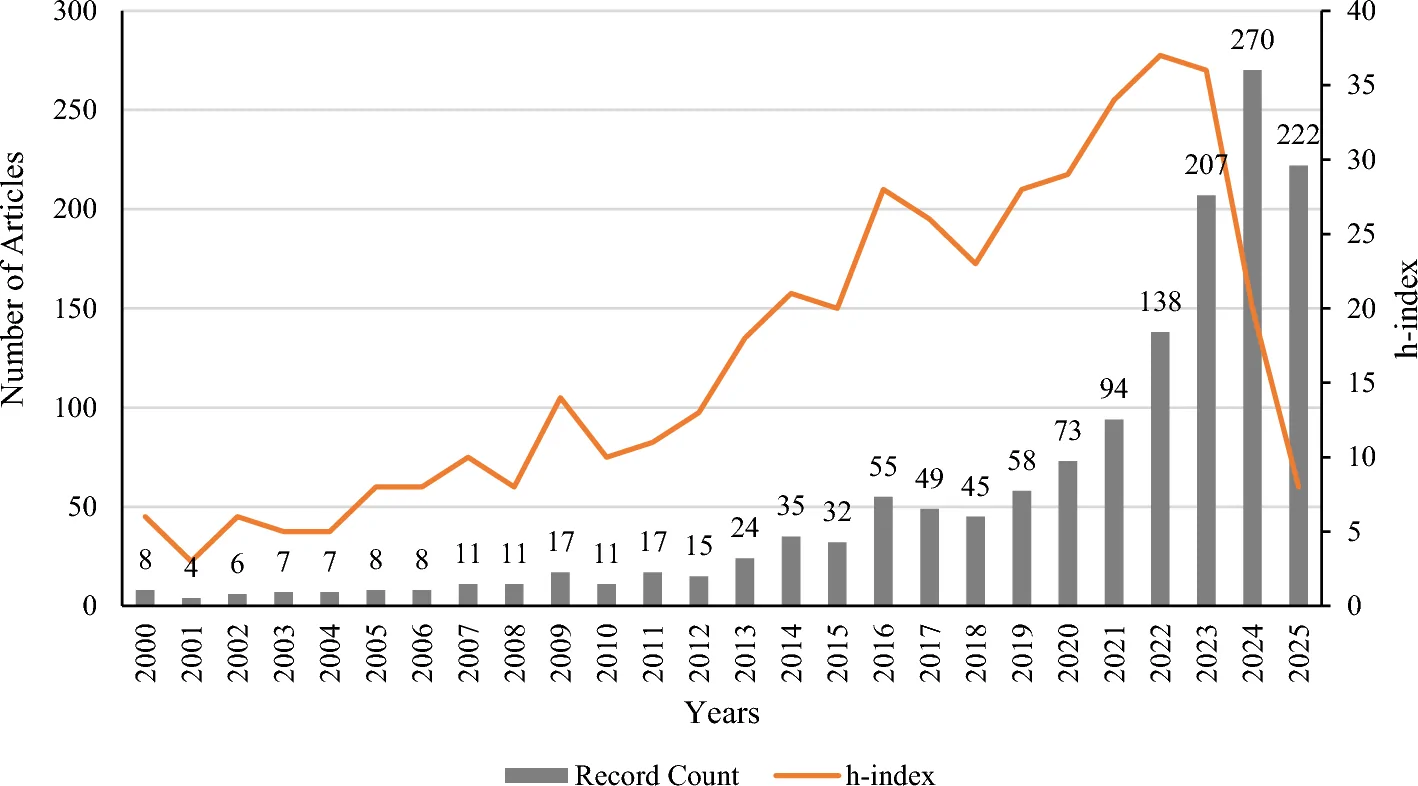
Annual number of publications and corresponding h-index for blue hydrogen production

Figure 6 shows the h-index trend for Blue Hydrogen Production between 2000 and 2025. It highlights how research impact has evolved alongside publication growth. In the early years from 2000 to 2012, the h-index remains low and somewhat irregular which reflects the limited number of papers and the fact that citations were concentrated in only a few influential studies. From 2013 onward, as publication activity increased, the h-index began to rise steadily. This means that more articles were consistently crossing the citation threshold. The index reached a strong and sustained level between 2017 and 2021 where that papers from this period gained recognition and were widely cited within the research community. The highest point occurs for last five years when both the quantity and influence of publications align and slowly getting peak in research impact.

**Fig. 7:**
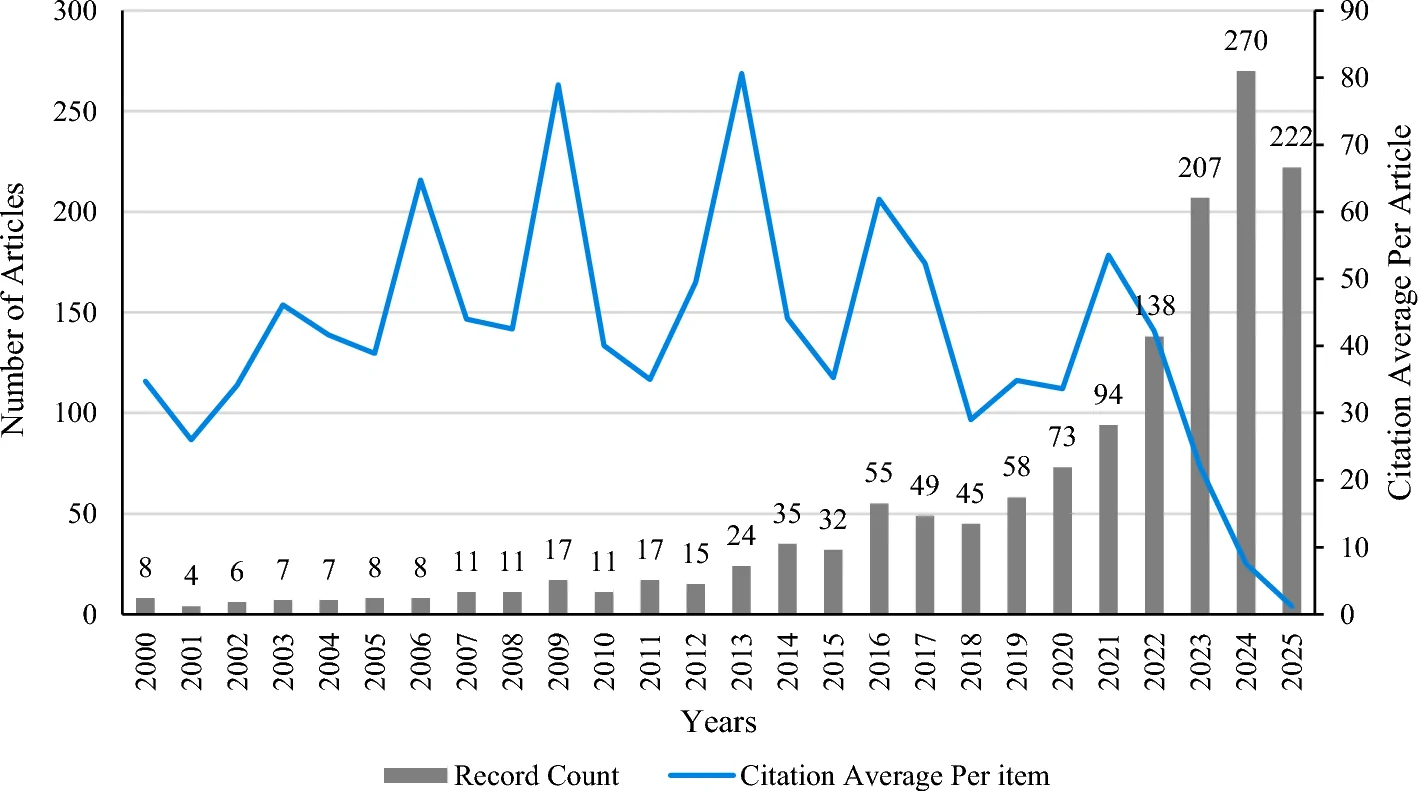
Annual number of publications and average citations per article on blue hydrogen Production

Figure 7 shows the trend of average citations per article on Blue Hydrogen Production. The trend changed substantially as the field developed. In the early 2000s, the average citation count per article was high and irregular because few papers had been published. These early studies also had more time to accumulate citations. As publication numbers began to increase in Technological expansion and CCS integration phase, the average citations per paper became more moderate. The periodic rises and falls indicate that highly influential reviews and key research papers was published as well as more routine studies. From 2020 onward, the average began to gradually decline while the overall number of publications surged, which indicate that citations were being distributed across a much-spread body of research.

Across all three figures, publications grow slowly and then rise gradually to a peak, the h-index reaches its own high after 2020. This shows that impact follows output after time to accrue citations. As research volume increases the average citations per article drift downward from the mid-2010s and drop after 2020, a classic dilution effect where many new papers share citations and have not yet been cited enough. A critical observation is that last five years combine high output with a sharp h-index and per-paper citation dip, which likely reflects short citation windows rather than weaker research. These years might show different results when revisited later.

**Fig. 8:**
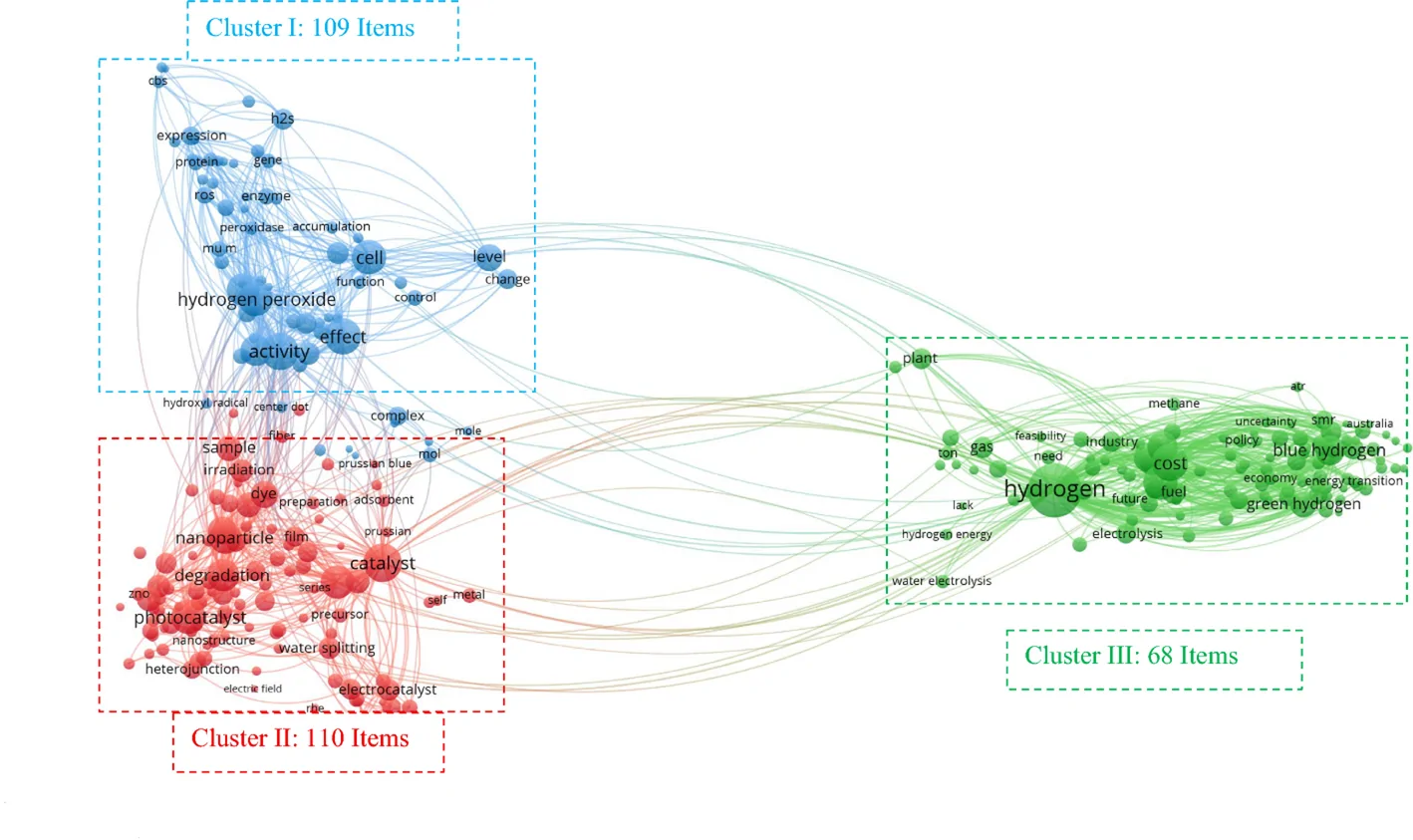
Term co-occurrence map of “blue hydrogen production” using VOSviewer (2000–2025) (minimum term occurrences = 20)

In Fig. 8, a bibliometric analysis was conducted using the VOSviewer version 1.6.20 to generate a keyword co-occurrence network. Dataset was collected from the WOSCC and records were exported with full metadata, including author keywords and Keywords Plus. The exported file was then imported into VOSviewer and a map was created based on bibliographic data. A co-occurrence analysis was selected with keywords defined as the unit of analysis, and the full counting method was applied so that each occurrence of a keyword was considered equally. Minimum occurrence of 20 was set to ensure the relevance of the terms and reduce noise. As a result, 478 keywords meeting the criteria out of which VOSviewer found 287 keywords for visualization. A network map was generated in which node size represented the frequency of keyword occurrence, colors indicated cluster, and thickness of connecting lines reflected the strength of co-occurrence relationships. This approach allowed the identification of research themes and the visual presentation of their interconnections within the field.

Three distinct clusters were identified in the analysis, as shown in Fig. 8. The first cluster is the red cluster consisting of 110 words. This cluster shows the photocatalysis, nanomaterials, and water splitting. Frequently occurring terms such as photocatalyst, degradation, nanoparticle, electrocatalyst, heterojunction, and water splitting highlight the strong research focus on advanced catalytic systems for hydrogen production and environmental remediation. This cluster represents the material science and catalytic innovation domain, where studies concentrate on synthesizing novel nanostructures, improving photocatalytic efficiency, and developing sustainable methods for hydrogen generation. The second cluster is the green cluster consisting of 109 words. This cluster captures research directly related to hydrogen energy systems and applications. Key terms include hydrogen, green hydrogen, blue hydrogen, cost, policy, economy, industry, feasibility, and water electrolysis. This cluster reflects the energy transition and systems integration perspective, where hydrogen is positioned as a clean energy carrier within broader industrial and economic contexts. Discussions in this cluster often extend to techno-economic assessments, renewable energy integration, and the policy frameworks necessary to accelerate hydrogen adoption.

The third cluster is blue cluster consisting of 68 words and shows terms associated with biological and chemical processes such as hydrogen peroxide, H₂S, cell, enzyme, gene expression, activity, and effect. This cluster highlights the biochemical and environmental pathways of hydrogen-related processes, including microbial hydrogen production, enzymatic activity, and oxidative mechanisms. It underscores the growing interest in exploring hydrogen generation and utilization from a biological and molecular science perspective, linking hydrogen studies with life sciences and environmental control.

**Fig. 9:**
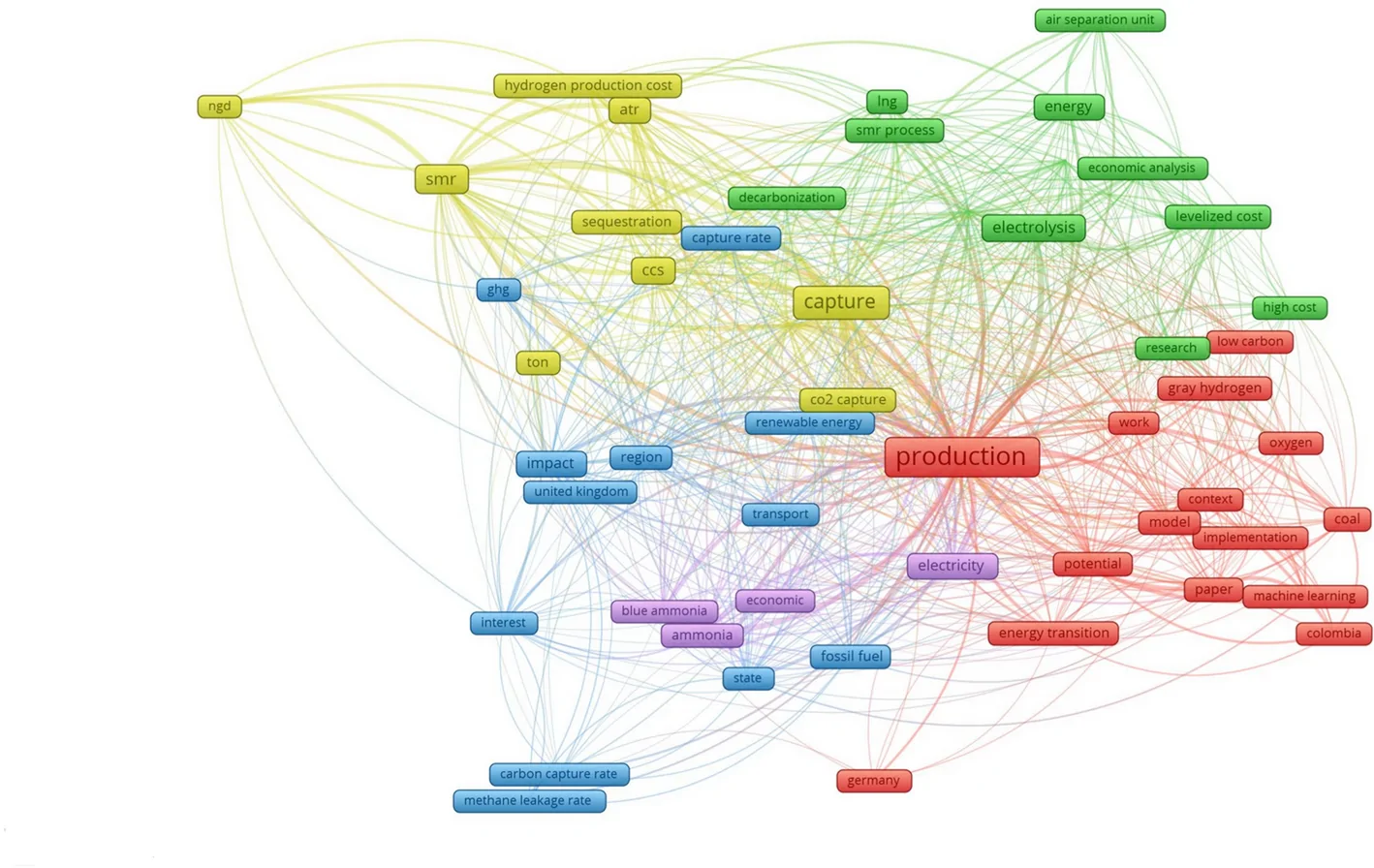
Term co-occurrence map of “blue hydrogen production with carbon capture” using VOSviewer (2000–2025) (minimum term occurrences = 5)

A second analysis was performed using the VOSviewer with the more specific query “Blue Hydrogen Production Carbon Capture” from the same WOS Dataset of only 166 articles and the result is shown in Fig. 9. This narrower search retrieved a smaller dataset that focused publications explicitly linking hydrogen production with carbon capture processes. Due to the reduced dataset size, a lower minimum occurrence (number of occurrences=5) threshold was applied to capture emerging but less frequent terms, while relevance scoring was used to highlight the most meaningful keywords. This methodological distinction allowed comparison between the broader thematic coverage of blue hydrogen research and the more focused cluster structure of studies integrating carbon capture technologies. Figure 9 shows a concentrated view where terms such as production, capture, CCS, electrolysis, and cost dominate the clusters. This highlights strong connections between hydrogen production pathways, carbon capture technologies, and economic considerations. The map is relatively focused, showing how blue hydrogen research is directly tied to decarbonization, capture rates, and system-level implementation.

### Meso-level analysis

Table 1 presents the top 10 institutions in blue hydrogen research. Among 216 contributing institutions, only 16% have published more than 10 articles, indicating a relatively concentrated research landscape. The Chinese Academy of Sciences (CAS) leads with 50 publications, 2010 citations, and the highest h-index (20), demonstrating both productivity and global impact. The Egyptian Knowledge Bank ranks second, with 27 articles, 569 citations, and an h-index of 14. It is followed by the Indian Institute of Technology System, with 24 articles and an h-index of 9, and the Council of Scientific and Industrial Research, with 23 articles and an h-index of 10. The University of California System stands out for its high citation impact per article and strong h-index (18) despite fewer publications.

**Table 1:** Top 10 influential institutions

Rank	University/Institution	Country (or Region)	Number of Articles	Number of cited articles	Total Citation	Citations Per Article	Citations Per Cited Article	h-index
1	Chinese Academy of Sciences	Peoples Republic of China	50	44	2010	40.20	45.68	20.0
2	Egyptian Knowledge Bank	Egypt	27	22	569	21.07	25.86	14.0
3	Indian institute of Technology System	India	24	22	439	18.29	19.95	9.0
4	Council of Scientific and Industrial Research (CSIR)	India	23	19	408	17.74	21.47	10.0
5	University of California System	United States of America	23	22	1326	57.65	60.27	18.0
6	French National Centre for Scientific Research (CNRS)	France	22	20	617	28.05	30.85	12.0
7	King Saud University	Saudi Arabia	22	17	495	22.50	29.12	10.0
8	South China University of Technology	Peoples Republic of China	19	18	254	13.37	14.11	10.0
9	Russian Academy of Sciences	Russia	17	17	417	24.53	24.53	10.0
10	State University System of Florida	United States of America	14	13	387	27.64	29.77	8.0

The French National Centre for Scientific Research (CNRS) contributed 20 articles and achieved an h-index of 12. King Saud University contributed 22 articles with an h-index of 10, while South China University of Technology published 19 articles with the same h-index. The Russian Academy of Sciences and the State University System of Florida also made notable contributions. Overall, CAS and the University of California System emerge as leaders in both productivity and influence.

**Fig. 10:**
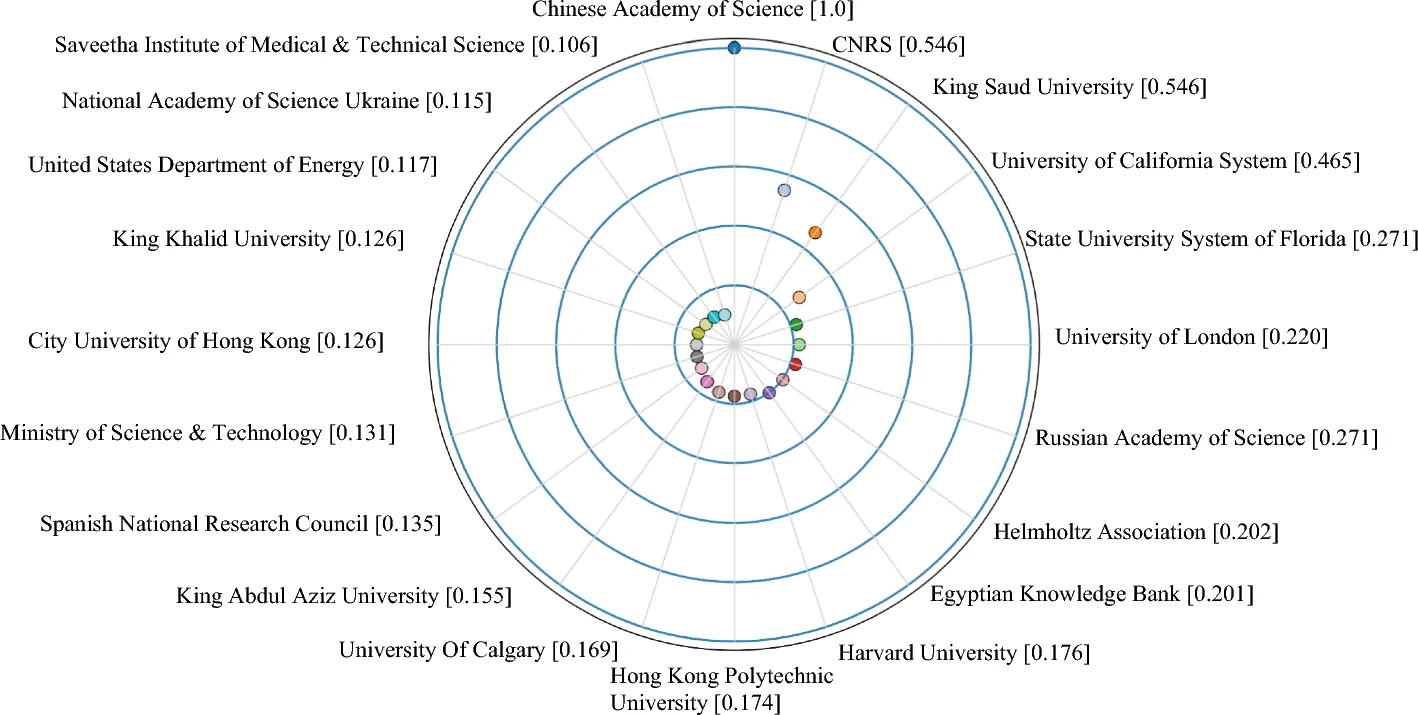
Centrality Wheel of Institutions (Normalized Values)

Figure 10 complements Table 1 by mapping institutional collaboration using normalized betweenness centrality (0–1), which reflects each institution’s brokerage role within the co-authorship network. CAS again stands out, positioned on the outer ring (centrality = 1.00), indicating it not only produces extensively but also acts as a key connector linking research groups and thematic clusters. The University of California System, King Saud University, and CNRS also occupy outer positions, suggesting strong international bridging capacity. In contrast, institutions such as IIT System, CSIR, the Russian Academy of Sciences, and the State University System of Florida, while productive, appear closer to the inner rings. This pattern implies more concentrated or regionally focused collaboration networks rather than broad, network-spanning roles. The Egyptian Knowledge Bank, despite ranking second in output, shows moderate centrality, indicating strength in dissemination but less influence as a structural broker.

The network also highlights influential institutions not listed among the top ten by publication count. Harvard University, the Helmholtz Association (Germany), Hong Kong Polytechnic University, the University of London, the U.S. Department of Energy, and Spain’s CSIC appear structurally central. Although they may publish fewer papers, their higher betweenness positions indicate important roles in connecting otherwise separate research communities. This finding underscores that impact arises from two complementary strategies: publication scale and strategic network positioning. Methodologically, the institutional collaboration network was constructed from co-authorship data, where nodes represent institutions and edges represent shared publications, weighted by frequency. Institutional names were standardized before analysis. Centrality metrics including degree, strength, closeness, eigenvector centrality, PageRank, and betweenness were computed in R. Betweenness centrality was selected for ranking and normalized using min–max scaling to ensure comparability. The centrality wheel visualization was plotted in polar coordinates with radial positions reflecting normalized betweenness and concentric rings (0.00–1.00) illustrating relative brokerage strength.

### Macro level analysis

**Fig. 11:**
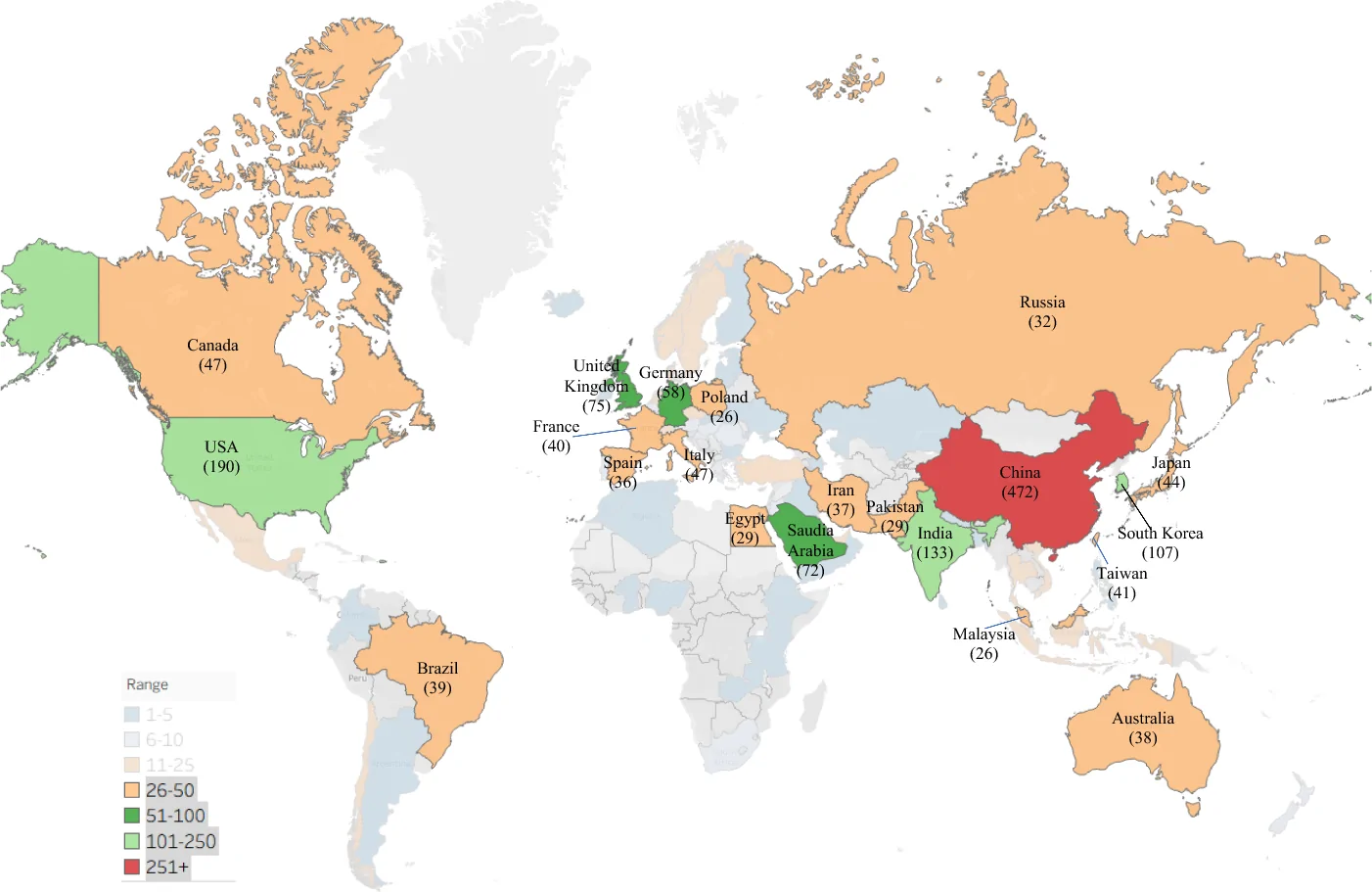
Geographical Distribution of Countries Publishing More Than 25 Articles on Blue Hydrogen Production

There were 92 countries involved in blue hydrogen production research since beginning of this century. Figure 11 shows geographical distribution of 21 Countries which have published more than 25 articles in last 25 years shows using data from the WOSCC and visualized in Tableau 2025.2. Peoples Republic of China clearly takes the leading position with 472 articles and placed it in the highest range of publications. The United States ranks second with 190 articles and India follows with 133 articles. Both fall into the 101–250 publication range. South Korea (107 articles) also belongs to this range. China, United States and India altogether published 795 articles which is 55.51% of the total Articles published by all the countries in the world. If we also include South Korea. The top 04 Countries have 63% research coverage and the rest of the world has 37%. These four countries each having more than 100 published articles has the leading position in blue hydrogen production research field.

A second group of countries demonstrates strong but secondary contributions are in the range of 51 to 100 publications. These include the United Kingdom with 75, Saudi Arabia with 72, and Germany with 58. The United Kingdom and Germany highlight the consistent role of European research and Saudi Arabia’s presence signals the Middle East’s determination to diversify its energy portfolio beyond oil and gas. These mid-level leaders are critical because they bridge advanced research ecosystems and resource-based economies. A larger cluster of countries falls into the 26 to 50 publication range. This group is diverse and developed economies such as Canada, Italy, Japan, France, and Australia along with emerging economies such as Brazil, Iran, Malaysia, Egypt, and Pakistan. Their involvement suggests that blue hydrogen is not only limited to high-income nations but also attracting broad participation from countries seeking to secure future energy resilience and reduce carbon emissions. These countries may not yet match the scale of global leaders, but their presence underlines the global spread of hydrogen-related research. Countries contribute fewer than 25 articles, including several in Europe, Southeast Asia, and the Middle East. Their involvement is modest but reflects the growing international recognition of blue hydrogen’s importance. The global map therefore reflects both concentration and diversification, showing that although a few nations drive most of the research, blue hydrogen is becoming a shared international priority.

Table 2 highlights the top cited most cited articles in blue hydrogen production based on total citations. Peoples Republic of China is the clear leader. It appears in the darkest color class on the map and tops Table 2 with 472 articles, 12,234 citations, and the highest h-index of 55. The United States, India, and South Korea form the next tier on the map (101–250 class). Table 2 shows their h-indices 48, 31, and 34 respectively. South Korea’s h-index (34) is higher than India’s despite fewer papers which implies higher average impact and consistent with its higher citations per article (39.81 vs 25.76). The 51–100 class on the map matches three of the top-ten for the United Kingdom (75 papers, h-index 29), Saudi Arabia (72 papers, h-index 20), and Germany (58 papers, h-index 24). Saudi Arabia is the only Middle Eastern country in the top-ten and shows a solid publication base although its citations per article (18.35) are lower than European peers.

**Table 2:** Top 10 Productive and influential countries & regions

Rank	Country	Region	Number of articles	Not Cited	Number of cited articles	Total Citation	Citation per article	Citation per cited articles	h-index
1	Peoples Republic of China	East Asia	472	55	417	12,234	25.92	29.34	55
2	United States of America	North America	190	13	177	8773	46.17	49.56	48
3	India	South Asia	133	15	118	3426	25.76	29.03	31
4	South Korea	East Asia	107	16	91	4260	39.81	46.81	34
5	United Kingdom	Europe (Western)	75	9	66	2717	36.23	41.17	29
6	Saudi Arabia	Middle East (Asia)	72	11	61	1321	18.35	21.66	20
7	Germany	Europe (Western)	58	9	49	2572	44.34	52.49	24
8	Canada	North America	47	5	42	3396	72.26	80.86	18
9	Italy	Europe (Southern)	47	3	44	1894	40.30	43.05	19
10	Japan	Asia (East Asia)	44	6	38	1375	31.25	36.18	16

Canada, Italy, and Japan appear in the 26–50 map class and occupy 8th, 9th and 10th position in Table 2. Canada records the highest citations per article (72.26) and per cited article (80.86) among the top-ten which shows strong impact per study even with fewer publications. Italy also shows citation efficiency by 40.30 citations per article. Japan’s h-index (16) and citation rates are lower than Italy’s, reflecting more moderate influence. Regionally, the map highlights an Asia centered research leadership by China, South Korea, India, Japan. On the other hand, United States and Canada are ahead in North America, and United Kingdom, Germany, Italy are consistent contributors in Europe. The Middle East is represented by Saudi Arabia. Brazil is the main South American presence just outside the top ten.

### Potential sources publishing research on blue hydrogen production

Table 3 illustrates the top ten sources publishing research on blue hydrogen production based on the number of articles, total citations, and average citations per article. Among these, the International Journal of Hydrogen Energy has publishing source with 131 articles with an average of 41.95 citations per article. The Journal of Cleaner Production and the Chemical Engineering Journal follows with 27 articles and 23 articles which contributes 660 and 429 citations respectively. In terms of citation impact, Journal of Colloid and Interface Science shows highest citation rate per article (51.77). The Energy Conversion & Management has 49.62 and the Journal of Material Chemistry has 44.07. Among emerging sources, Energies published 23 articles with 376 citations with an average of 16.35 citations per article. The New Journal of Chemistry and RSC Advances contributed 16 and 15 articles respectively. Looking at publisher influence, Elsevier dominates, accounting for 6 out of the 10 journals, the Royal Society of Chemistry contributes 03 journals, and MDPI represents one source. Overall, the top ten journals collectively produced 379 articles, received 14,946 citations, and reported an average of 39.4 citations per article.

**Table 3:** Top sources publishing research on blue hydrogen production based on total number of articles

Rank	Publication source	Research area/category	Number of articles	Total citation	Citation per article	Publisher	IF	CS
1	International Journal of Hydrogen Energy	Energy Engineering & Power Technology; Fuel Technology; Renewable Energy, Sustainability & the Environment	131	5496	41.95	Elsevier	8.3	13.3
2	Journal of Cleaner Production	Environmental Science; Industrial & Manufacturing Engineering; Renewable Energy	27	660	24.44	Elsevier	11.1	15.1
3	Chemical Engineering Journal	Chemical engineering Chemistry, Industrial and Manufacturing Engineering	23	429	18.65	Elsevier	7.1	12.4
4	Energies	Energy Engineering & Power Technology; Electrical & Electronic Engineering; Renewable Energy, Sustainability & the Environment	23	376	16.35	MDPI	3.2	5.8
5	Energy Conversion & Management	Energy Engineering & Power Technology; Fuel Technology; Renewable Energy, Sustainability & the Environment	21	1042	49.62	Elsevier	11.5	18.5
6	New Journal of Chemistry	Chemistry; Inorganic Chemistry; Materials Science	16	238	14.88	Royal Society of Chemistry	3.9	6.7
7	Applied Energy	Energy Engineering & Power Technology; Fuel Technology; Renewable Energy, Sustainability & the Environment	15	242	16.13	Elsevier	11.4	20.4
8	Journal of Material Chemistry A	Materials science, especially for energy and sustainability, Encompasses synthesis, properties	15	661	44.07	Royal Society of Chemistry	9.5	16.7
9	RSC Advances	Chemical Engineering (miscellaneous) (Q1), Chemistry (miscellaneous) (Q2), Covers all aspects of Chemical sciences	15	269	17.93	Royal Society of Chemistry (United Kingdom)	4.6	5.9
10	Journal of Colloid and Interface Science	Colloid and interface science fundamentals, Applications including colloidal materials, nanomaterials	13	673	51.77	Academic Press (an imprint of Elsevier)	9.7	9.7

**Fig. 12:**
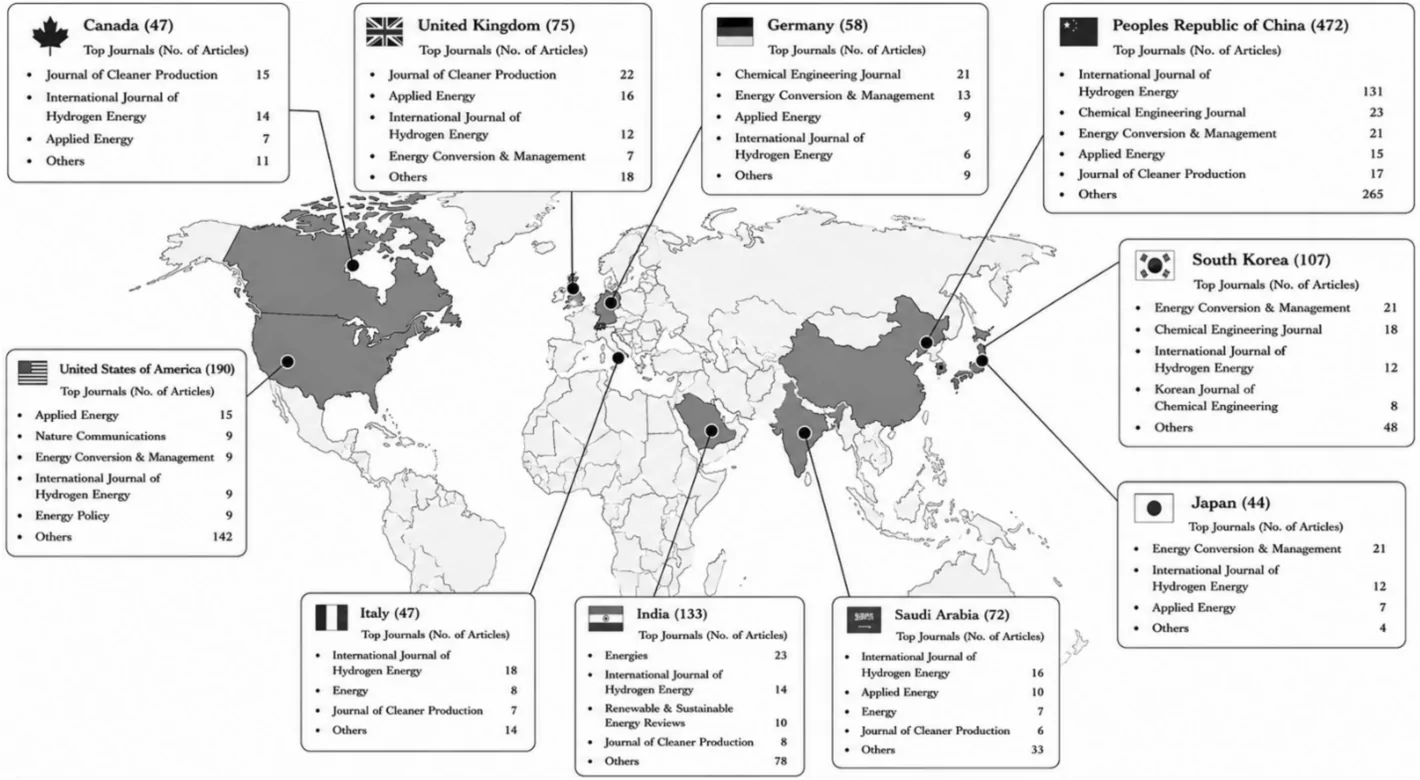
Global distribution of publication sources and regional structure

Figure 12 reveals regional differences in the distribution of publication sources and publication structures among leading countries involved in blue hydrogen research. The Peoples Republic of China demonstrates strong publication concentration in engineering and process-oriented journals such as International Journal of Hydrogen Energy and Chemical Engineering Journal, reflecting emphasis on SMR, catalyst development, and CCS integration. The United States shows representation in multidisciplinary and energy-policy-related journals, highlighting research focus on hydrogen hubs, commercialization, techno-economic assessment, and industrial deployment. European countries such as the United Kingdom and Germany demonstrate publication structures strongly associated with industrial decarbonization, CCS transport infrastructure, and energy transition studies through journals including Applied Energy and Journal of Cleaner Production. Canada exhibits strong publication activity in sustainability-oriented and life-cycle assessment research, particularly related to low-carbon hydrogen systems and geological CO₂ sequestration. Asian countries including South Korea and Japan emphasize process optimization, advanced hydrogen systems, and energy integration studies. These findings demonstrate that the publication structure of different countries is influenced by regional industrial priorities, energy transition policies, CCS infrastructure development, and national hydrogen deployment strategies.

## Research frontier in subject category

**Fig. 13:**
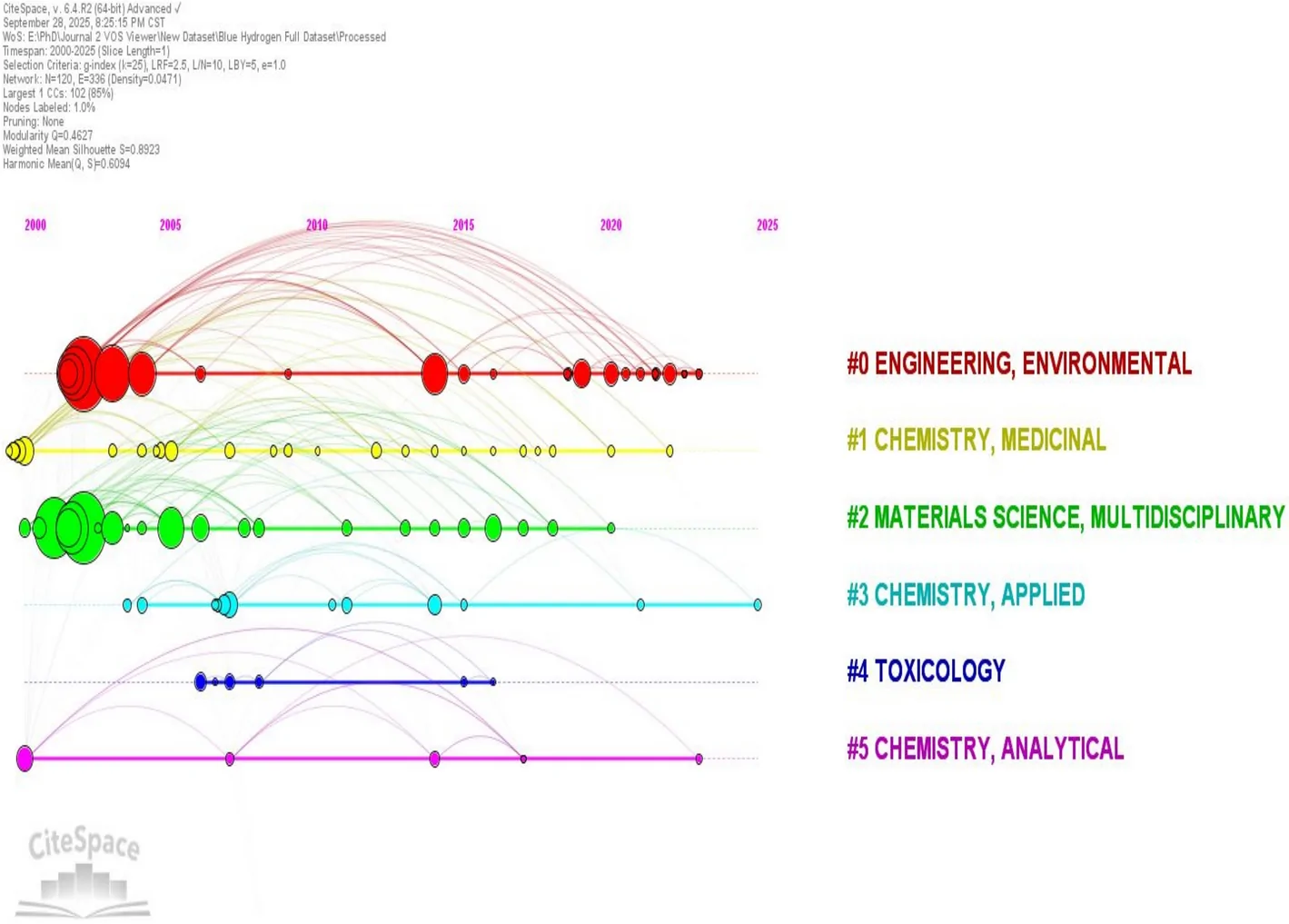
Subject category evolution timeline

Figure 13 illustrates the subject category timeline cluster map generated using CiteSpace (v6.4.R2) based on WOSCC data from 2000 to 2025, with one-year time slices to capture annual developments. These phases are consistent with the broader developmental stages identified in publication trend and topic evolution analyses. The category node type was selected to track disciplinary contributions over time. Node selection was controlled using the g-index (k = 25) with parameters (LRF = 2.5, LBY = 5, e = 1.0), ensuring inclusion of influential categories while maintaining network clarity. Clusters were labeled using the log-likelihood ratio (LLR) method, resulting in six dominant groups: Engineering & Environmental, Chemistry Medicinal, Materials Science Multidisciplinary, Chemistry Applied, Toxicology, and Chemistry Analytical.

In the timeline visualization, each horizontal line represents a cluster, with nodes indicating subject prominence and links reflecting co-occurrence or citation relationships across years. Chemistry Analytical (Cluster #5) appears as early as 2000, demonstrating the foundational importance of analytical techniques in validating hydrogen-related experiments. During the 2010–2018 period, Engineering & Environmental (Cluster #0) and Materials Science (Cluster #2) expanded significantly, signaling a shift from fundamental chemical studies to applied environmental solutions and advanced material innovations. This phase aligns with growing global emphasis on CO₂ mitigation and clean energy technologies. In the most recent stage (2018–2025), Toxicology (Cluster #4) and Applied Chemistry (Cluster #3) gained visibility, reflecting increased attention to safety, risk assessment, and industrial scalability as hydrogen technologies approach real-world deployment. The overlap between Applied Chemistry and Materials Science highlights strong cross-disciplinary integration, particularly in process optimization and material-based system design. The analysis identifies three evolutionary phases. Research conducted between 2000 and 2010 was primarily chemistry-driven. From 2010 to 2018, the field expanded into environmental engineering and materials innovation. Between 2018 and 2025, greater attention to safety, risk, and interdisciplinary applications indicated the maturation of hydrogen research.

### Factorial analysis

#### Trend topic

**Fig. 14:**
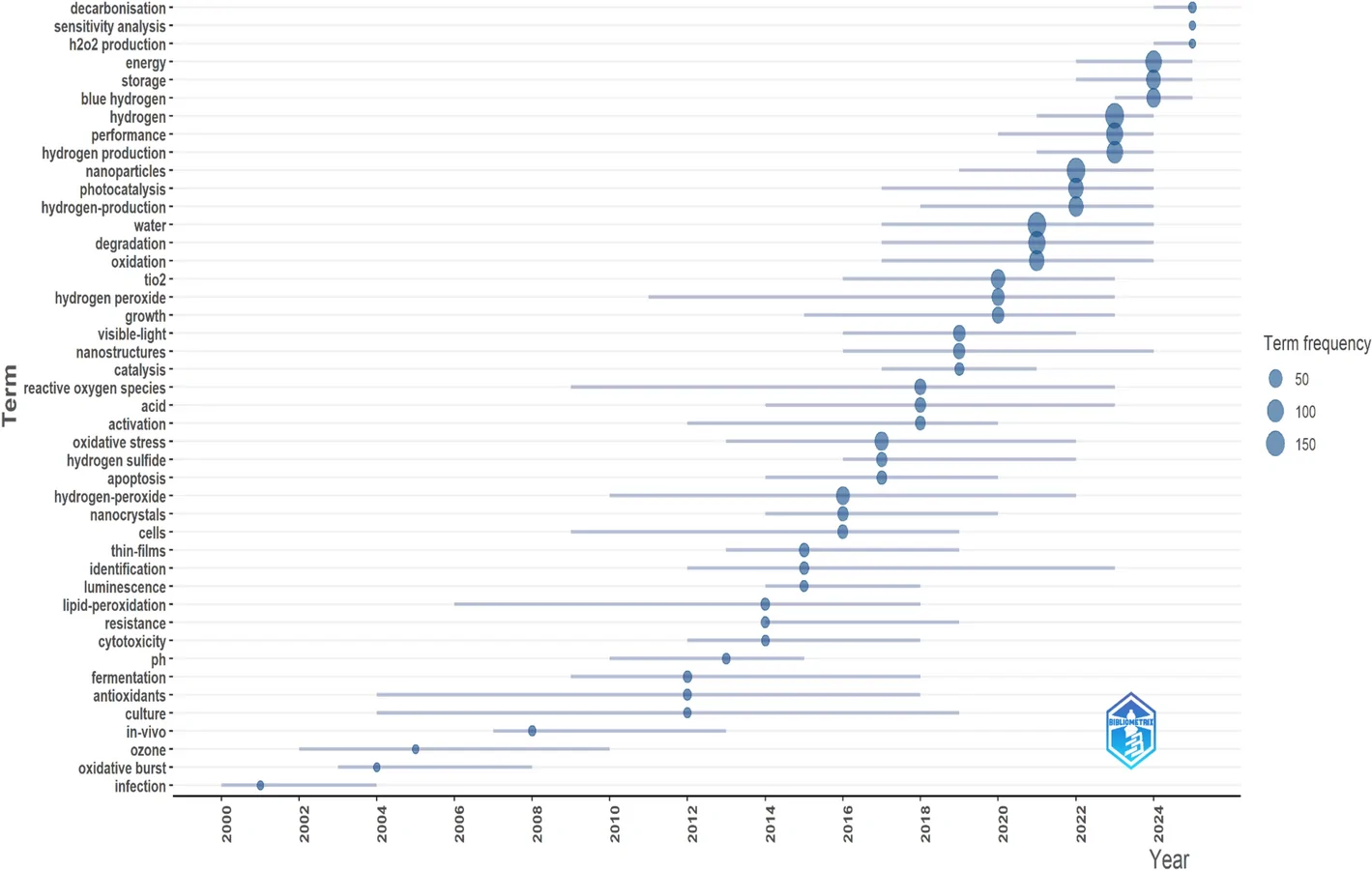
Topic trends in blue hydrogen research over the last 25 years

Although the study focuses on blue hydrogen production, the trend-topic analysis captures the broader research themes emerging from the retrieved literature. In metrological analysis, the research frontier was studied by the Bibliometrix function in the R language to obtain the trend of tropic across time. Figure 14 presents the trend between 2000 and 2025. Before 2010, there were only a few keywords such as “ozone” and “infection” with less than 10 occurrences. During the technological expansion phase between approximately 2010 and 2017, the research landscape expanded to include more technical terms. Keywords such as photocatalysis, Titanium oxide, oxidation, and reduction gained increasing attention. These terms indicate that hydrogen research at this stage was oriented towards catalytic processes, nanomaterials, and reaction mechanisms. Hydrogen production also began to appear more frequently, reaching nearly 100 occurrences in this period. A major transition toward interdisciplinary and sustainability-oriented research emerged after 2017–2018, when research output grew significantly and the focus of investigation broadened. High-frequency terms included hydrogen appeared 150 times, energy appeared 96 times, nanoparticles appeared 141 times, and performance appeared 105 times. These terms reflect the emphasis on scaling hydrogen technologies and optimizing their efficiency. The emergence of blue hydrogen as a keyword appeared 62 times. This demonstrates the consolidation of this theme into a recognized research frontier. At the same time the word “hydrogen production” was found 95 times and storage came up 96 times. This scenario illustrates the technical challenges and system-level issues being investigated.

For the last five years, there is a rapid growth sustainability-oriented terms such as decarbonization, sensitivity analysis, and LCA. These indicate a clear transition from laboratory-based research to broader system-level evaluations of environmental impact and policy relevance. Moreover, frontier topics such as green hydrogen and renewable energy emerged alongside Blue Hydrogen. Overall, the trend shows that Blue Hydrogen research has moved from scattered chemical studies in the early 2000s to interdisciplinary investigations in the 2020s. The combination of high-frequency keywords such as hydrogen, nanoparticles, energy, and blue hydrogen with sustainability-oriented terms like decarbonization and LCA confirms that the field is now both technologically advanced and policy driven. Future research frontiers will increasingly focus on optimizing production efficiency, integrating renewable energy, and evaluating environmental and economic impacts through system-level analysis.

#### Keyword dendrogram

In this study, a hierarchical clustering approach was applied to identify the structural relationships among the most frequently occurring keywords in the dataset. The dendrogram was drawn using the factorial analysis function in Bibliometrix (R language), which groups terms based on their co-occurrence patterns in the literature. A distance measure was first calculated between keywords, followed by the application of a clustering algorithm that progressively merges related terms into broader groups. The dashed vertical line in the figure indicates the chosen cut-off level for identifying major clusters.

**Fig. 15:**
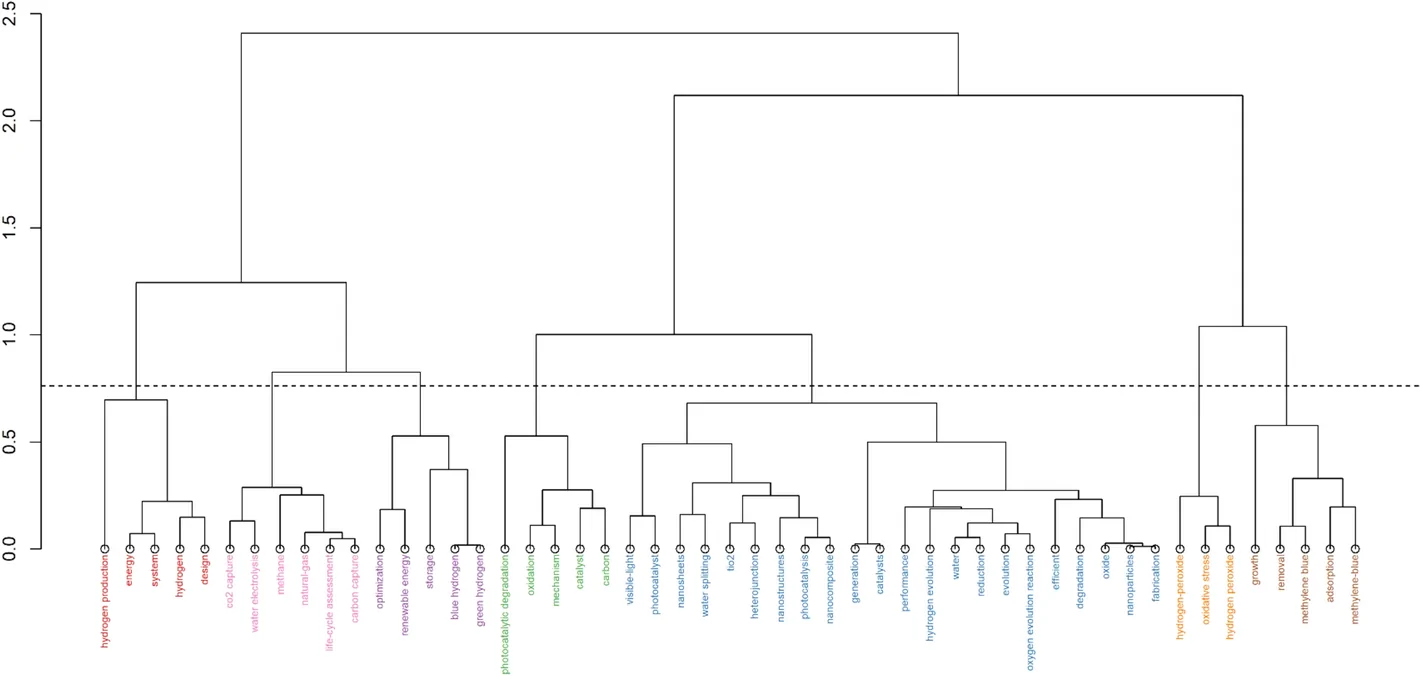
Keyword dendrogram of blue hydrogen research

Figure 15 presents the topic dendrogram derived from 1432 documents, revealing four principal clusters of research themes. The first cluster (shown in red) is centered on “hydrogen production,” “system,” “design,” “energy,” and “CO₂ capture,” representing the engineering and system-level analysis of hydrogen technologies with emphasis on carbon capture and renewable energy integration. The second cluster (green and blue) includes terms such as “photocatalytic degradation,” “oxidation,” “nanostructures,” “TiO₂,” “photocatalysis,” and “hydrogen evolution,” reflecting the material science and photocatalytic research domain. The third cluster (orange) groups terms like “hydrogen peroxide,” “oxidative stress,” and “growth,” which are linked to environmental impacts, hydrogen–oxygen chemistry, and biological implications. Finally, the fourth cluster (purple and related terms) connects “renewable energy,” “storage,” “blue hydrogen,” and “green hydrogen,” pointing to studies on sustainable pathways and energy transition strategies. Overall, the dendrogram highlights how research on hydrogen is fragmented into system design and carbon capture, nanomaterial-driven photocatalysis, environmental and oxidative pathways, and renewable integration and hydrogen colors. These clusters confirm that hydrogen research is inherently interdisciplinary, spanning engineering, material science, environmental chemistry, and energy policy.

#### Multiple correspondence analysis for conceptual structure mapping

**Fig. 16:**
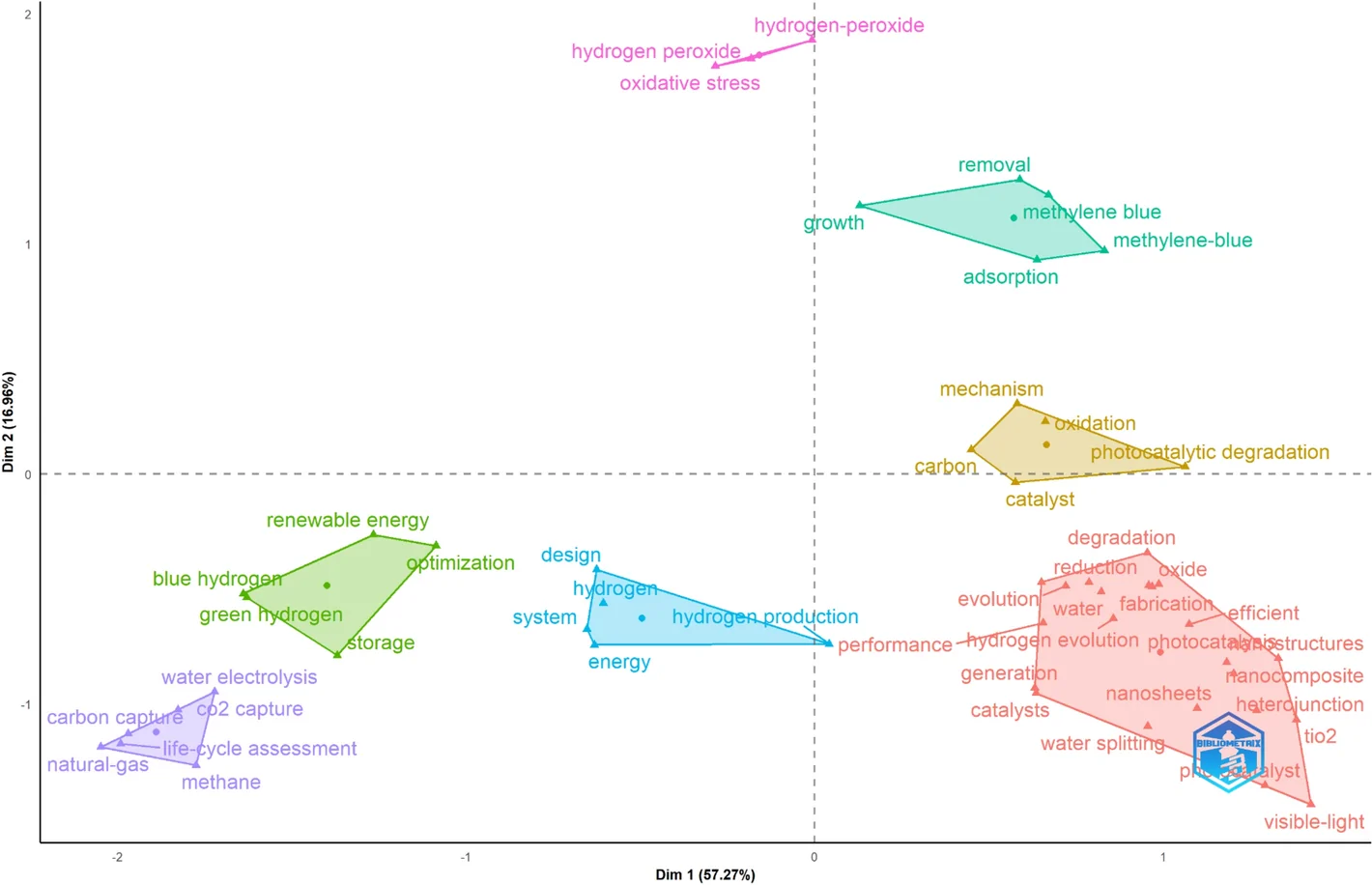
Conceptual structure map of hydrogen research keywords based on MCA

A factorial analysis using MCA in the Bibliometrix R package was conducted after filtering keywords and applying co-occurrence analysis. MCA positioned terms on a two-dimensional map, where Dim 1 (57.27% variance) captured the dominant thematic structure and Dim 2 (16.96%) provided complementary differentiation. Based on proximity and co-occurrence, six conceptual clusters emerged (Fig. 16). The blue cluster centers on photocatalyst, water splitting, TiO₂, nanostructures, and performance, representing photocatalytic hydrogen production and semiconductor materials research. The green cluster includes photocatalytic degradation, oxidation, catalyst, and carbon, reflecting environmental and pollutant-removal applications. The red cluster (hydrogen, hydrogen production, energy) captures core energy-transition themes. The violet cluster connects blue hydrogen, green hydrogen, renewable energy, storage, and optimization, emphasizing sustainable integration pathways. The pink cluster (natural gas, CO₂ capture, methane, and LCA) highlights SMR, CCS, and LCA research. The orange cluster (hydrogen peroxide, oxidative stress, adsorption, removal) reflects biomedical and environmental applications. Overall, the figure demonstrates hydrogen research’s multidimensional structure, spanning energy production, carbon management, renewable integration, materials science, and biomedical applications within the broader hydrogen economy.

### Bibliometric leadership and industrial deployment

Table 4 demonstrates the relationship between global bibliometric leadership in blue hydrogen research and the practical industrial deployment of Hydrogen production using a dataset of infrastructure projects published by IEA [44]. The comparison shows that countries with high publication output, citation impact, and strong collaboration networks also lead commercial blue hydrogen and CCS development. These countries include China, the United States, Canada, and the United Kingdom. It further highlights how research growth after 2020 aligns with increasing investments in hydrogen production capacity, CO₂ capture systems, industrial decarbonization strategies, and low-carbon energy transition initiatives.

**Table 4:** Relationship between global bibliometric leadership and recent industrial deployment

Country/Region	Bibliometric findings (2000–2025)	Hydrogen Production and Infrastructure Projects Database [44]					Research–Industry Connection
		Specific blue hydrogen / CCS project	Project status	Online year	Hydrogen capacity (kilo Ton per year)	CO₂ capture capacity (Ton per year)	
Peoples Republic of China	⇒ 472 articles, ⇒ h-index 55, ⇒ Highest global publication output	Sinopec CCS-integrated Hydrogen Projects	Announced / Expansion	2022	Large-scale expansion projects	Expanding CCS deployment	Strong publication growth aligns with rapid industrial hydrogen expansion and CCS investment
United States of America	⇒ 190 articles, ⇒ h-index 48, ⇒ High citation impact	ExxonMobil Baytown Hydrogen	Announced	2029	919.3	7,000,000	High-impact research supports commercialization of mega-scale blue hydrogen facilities
	⇒ Collaboration and industrial decarbonization research	ACE Complex, Louisiana — Phase 1 ACE Complex, Louisiana — Phase 2	Announced	Phase 1: 2029 Phase 2: Not specified	864.3	7,600,000	Research activity strongly linked with large-scale CCS-integrated hydrogen deployment
	⇒ Advanced CCS and hydrogen systems research	Eastern Louisiana Clean Hydrogen Complex	Announced	2029	689.9	5,000,000	Strong academic and industrial alignment in hydrogen hub development
Canada	⇒ 47 articles, ⇒ highest citation efficiency (72.26 citations/article)	Quest CCS	Operational	2015	300.0	1,100,000	Strong CCS expertise supports practical geological CO₂ storage deployment
	⇒ High-impact CCS and hydrogen integration studies	Blue but Better (Alberta)	FID / Construction	2028	548.0	3,000,000	Research supports Alberta hydrogen hub and low-carbon infrastructure
	⇒ Strong focus on sequestration and hydrogen systems	Linde Fort Saskatchewan	Planned	2029	1000.0	2,200,000	Existing sequestration infrastructure enhances industrial deployment potential
United Kingdom	⇒ 75 articles, ⇒ strong international collaboration network	H21 Leeds City Gate	Feasibility / Planning	2035	3033.4	15,000,000	Research trends strongly connected with industrial cluster-based hydrogen systems
	⇒ Strong CCS transportation and infrastructure studies	HyNet Northwest	Planning	Phase: 1 2027 Phase: 2 2030	810.0	6,200,000	Collaboration research supports integrated hydrogen and CO₂ transport systems
South Korea	⇒ 107 articles, ⇒ h-index 34, ⇒ high citation efficiency	Hydrogen Import & Ammonia Co-firing Projects	Developing	2030 (Policy Development Phase)	Emerging deployment	Developing CCS-linked infrastructure	Research supports national hydrogen transition and energy security strategies
Saudi Arabia	⇒ 72 articles, ⇒ growing hydrogen research output	NEOM Low-Carbon Hydrogen Project	Under Development	2027	Large export-oriented projects	Expanding low-carbon infrastructure	Research aligns with economic diversification and hydrogen export strategies
Germany	⇒ 58 articles, ⇒ strong European collaboration and CCS studies	Industrial Hydrogen + CCS Projects	Multiple Projects	2028–30 (multiple project timeline)	Medium-large deployment	Active CCS integration	Research emphasizes industrial decarbonization and hydrogen system integration
Netherlands	⇒ Active CCS and industrial systems integration research	Onyx Plant – Port of Rotterdam	Planned	2028	300.0	–	Strong linkage between hydrogen production and CO₂ transport/storage infrastructure
Norway	⇒ Strong collaboration-based CCS and offshore storage research	Northern Lights Blue Hydrogen Concepts	Emerging	2024	Export-oriented development	Offshore CCS expertise	Existing offshore CCS capability supports blue hydrogen commercialization
France	⇒ Strong institutional contribution and citation impact	Port Jérôme Hydrogen Project	Operational	2015	39.0	100,000	Institutional research supports industrial hydrogen deployment and CCS integration

## Collaboration analysis

This chapter looks at how researchers, institutions, and countries work together in this field. Collaboration analysis was conducted using VOSviewer at three levels co-authorship, institutional, and national providing insights into the structure of research relationships, distribution of scientific contributions, and degree of global connection. This approach helps identify the active contributors, the hubs of institutional productivity and leading countries driving the field. This also helps to understand the areas where collaboration remains limited. In the maps, the circle size shows how many papers they published, the line thickness shows how strongly they worked together, and the colors show changes over time.

### Collaboration of country

The network was generated in VOSviewer using co-authorship (countries as the unit of analysis) based on WOSCC data exported with full records and cited references. Full counting was applied so each co-authorship contributed equally. A minimum threshold of 10 documents per country reduced the network from 92 to 40 countries, and documents with more than 25-country co-authorship were excluded to avoid distortion. The network visualization displays node size proportional to publication output, link thickness representing collaboration strength (Eq. 6), and color clusters indicating collaboration groups and temporal evolution.

**Fig. 17:**
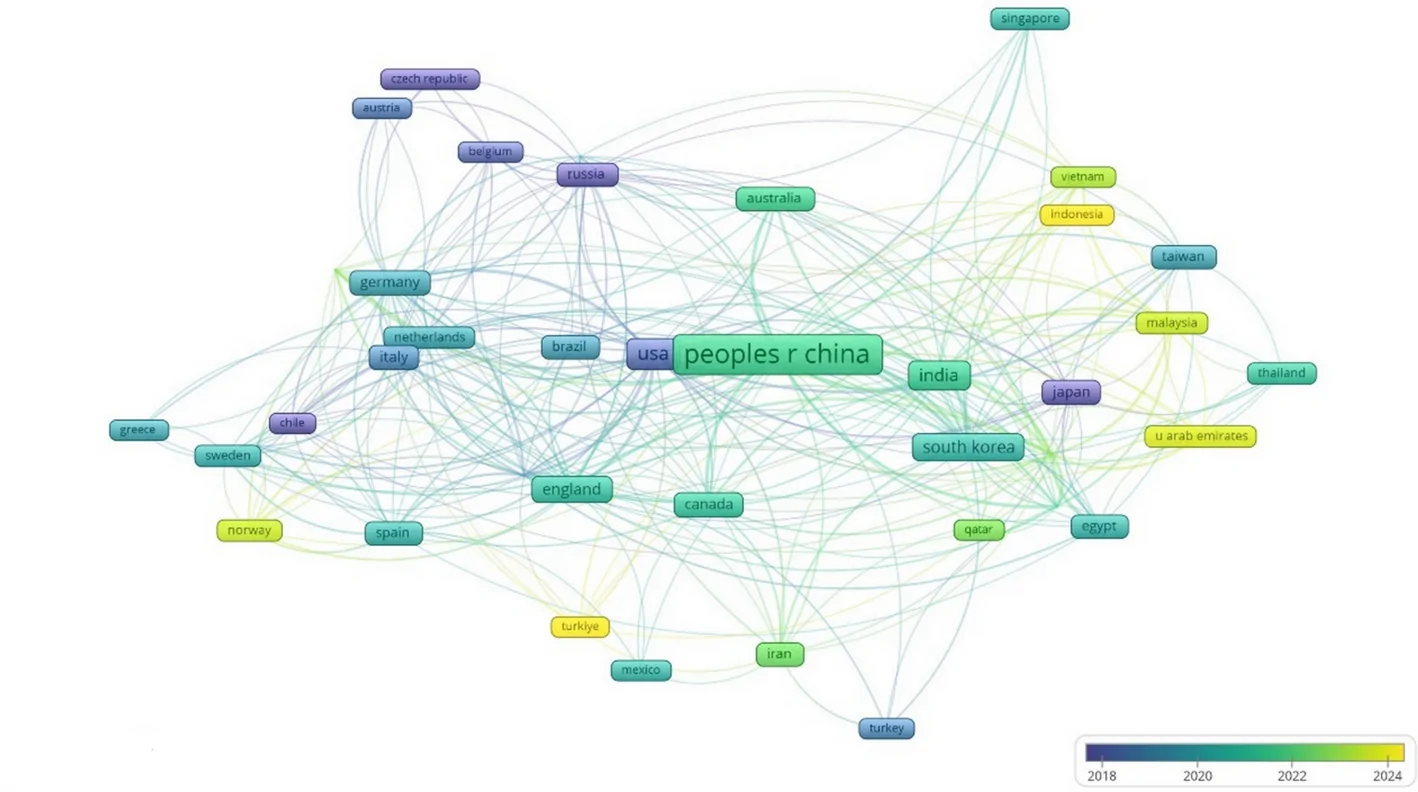
Network visualization map of global research collaboration (minimum of 10 publications per country)

Although 92 countries contributed to this field, Fig. 17 presents 40 countries meeting the threshold. Early activity (2018–2019) was led by Russia, Japan, Belgium, Austria, and the Czech Republic. Between 2020 and 2021, leadership shifted to China, the United States, Germany, India, South Korea, and England, with Brazil, Canada, and Australia increasing visibility. In the most recent phase, participation expanded to the United Arab Emirates, Malaysia, Indonesia, Vietnam, Norway, and Turkey, often collaborating with established leaders such as China. Overall, the field evolved from early European and Japanese engagement to dominance by major global players, followed by broader international expansion, particularly in the Middle East and Southeast Asia.

### Collaboration of organizations

Institutional collaboration in blue hydrogen research was analyzed using VOSviewer based on WOSCC data (full record and cited references). Full counting was applied so each institution received equal credit, and documents with more than 25 organizational co-authors were excluded to avoid distortion from large consortium projects. A minimum threshold of six publications per institution reduced the dataset from 1906 to 57 institutions; the largest connected component of 43 institutions was retained for coherent visualization.

**Fig. 18:**
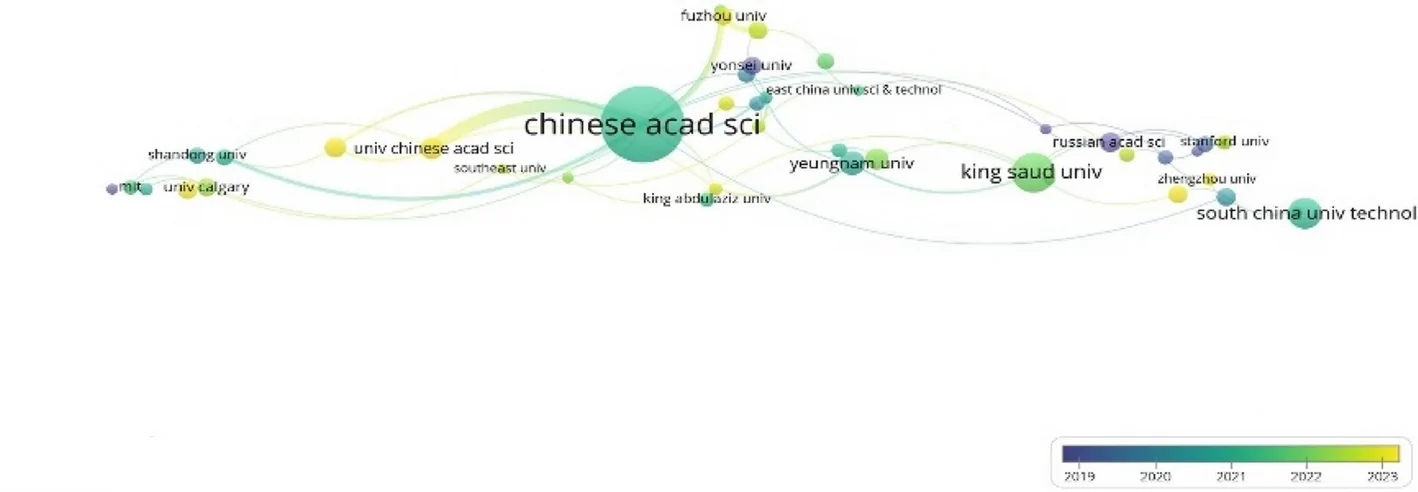
Network overlay visualization map of organizational collaboration (minimum of 6 publications per organization)

In Fig. 18, node size reflects publication output, link thickness indicates collaboration strength, and color clusters represent temporal evolution. The Chinese Academy of Sciences emerges as the most dominant institution, with high publication volume and extensive global collaboration. In earlier years, the Russian Academy of Sciences, MIT, and Stanford University were active but at smaller scales. Between 2020 and 2021, institutions such as the University of Calgary, Shandong University, and Yeungnam University expanded the collaboration network beyond China. Simultaneously, the University of Chinese Academy of Sciences and East China University of Science and Technology strengthened ties with the Chinese Academy of Sciences. In the 2022–2023 period, King Saud University, King Abdulaziz University, and South China University of Technology gained visibility, indicating growing Middle Eastern and South Chinese engagement alongside continued Chinese institutional leadership.

### Collaboration of researchers

The researcher collaboration network was constructed in VOSviewer to examine co-authorship patterns in blue hydrogen research (2000–2025) using WOSCC data exported with full records and cited references. Co-authorship was selected as the analysis type and authors as the unit of analysis, with full counting applied to weight each collaboration equally. A total of 7215 authors contributed to the field; applying a minimum threshold of two documents reduced this to 533 authors. Documents with more than 25 co-authors were excluded, and only the largest connected component (36 authors) was retained to ensure a coherent network.

**Fig. 19:**
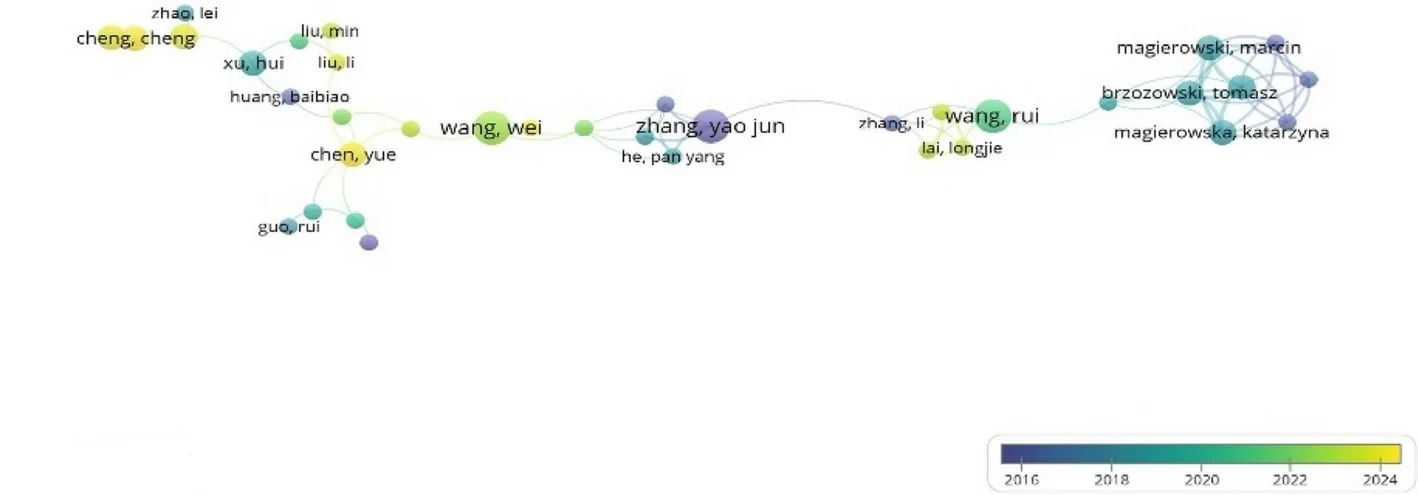
Network overlay visualization of researcher collaboration (minimum of 2 publications per researcher)

In Fig. 19, node size represents publication output, link thickness indicates collaboration strength, and colors reflect temporal evolution. Early contributors (pre−2018) such as Huang Baibiao, Zhang Yao Jun, and Guo Rui (blue/purple nodes) collaborated closely with Xu Hui and Liu Min. Between 2018 and 2020, Wang Wei and Chen Yue (green nodes) acted as bridges linking earlier and emerging groups. From 2021 to 2024, Cheng Cheng, Zhao Lei, and Wang Rui (yellow nodes) became prominent. On the European side, researchers such as Magierowski Marcin, Brzozowski Tomasz, and Magierowska Katarzyna (teal cluster) have shown strong collaboration activity in recent years.

## Discussion, implications and research outlook

In this section all the evidence has been integrated from the keyword networks in Figs. 8 and 9, the regional and subject-evolution analyses in Figs. 12, 13, 14, the thematic analyses in Figs. 15 and 16, the collaboration networks in Figs. 17 and 18. The topic-trend analysis identifies when major research themes emerged, the keyword dendrogram explains their hierarchical relationships, and the multiple correspondence analysis demonstrates their conceptual interconnections. Collectively, these findings indicate that blue hydrogen research has evolved from fragmented material- and process-level investigations into an increasingly integrated, system-oriented, and sustainability-driven field. Within this evolution, CCS is positioned as a transitional technology connecting conventional natural-gas-based hydrogen production with broader industrial decarbonization pathways. The principal findings and implications can be organized into four interconnected dimensions: techno-economic and technological performance, integrated environ.

### Techno-economic and technological performance

The keyword networks in Figs. 8 and 9 and the subject-evolution analysis in Fig. 13 show that “cost,” “production,” “capture,” “CCS,” and “energy” are central to blue hydrogen research. The keyword dendrogram in Fig. 15 further connects CO₂ capture with engineering and system optimization. These findings indicate that production cost cannot be separated from capture efficiency, energy consumption, compression, transportation, and storage requirements. Future studies should therefore assess hydrogen production and CCS as one integrated system. Technological research identified in Figs. 8 and 13, including advanced catalysts, nanomaterials, and process engineering, may be strengthened through artificial intelligence, digital twins, advanced control, and system dynamics modelling to improve yield, reduce energy penalties, and lower cost.

### Integrated environmental performance and renewable coupling

The research-topic trends in Fig. 14 and the thematic analyses in Figs. 15 and 16 show increasing attention to life-cycle assessment, sustainability, renewable energy, storage, and hybrid hydrogen systems. These results indicate that environmental performance cannot be evaluated only from the plant-level CO₂ capture rate. Upstream natural-gas extraction, methane leakage, residual emissions, energy use, transportation losses, and storage integrity must also be included. Consistent system boundaries and harmonized life-cycle assessment methods are therefore required. Figures 14 and 16 also connect blue hydrogen with green hydrogen, renewable energy, and optimization, supporting the assessment of renewable-assisted configurations. Future research should compare conventional blue hydrogen, renewable-assisted blue hydrogen, biomass-integrated systems, and green hydrogen using consistent technical, economic, and environmental indicators.

### Infrastructure, supply-chain resilience, and regional deployment

The geographical and regional analyses in Figs. 11 and 12 demonstrate that commercialization depends on access to hydrogen distribution systems, CO₂ pipelines, geological storage, industrial users, and shared infrastructure. Transportation options should be selected according to production scale, distance, geography, and market demand. The collaboration networks in Figs. 17, 18, 19 further show that blue hydrogen development requires coordination among countries, institutions, and researchers. Large projects may also face feedstock disruption, equipment failure, transportation constraints, storage limitations, and uncertain demand. Future research should apply scenario analysis, fault-tree methods, agent-based modelling, and resilience optimization. Because Figs. 11 and 12 reveal substantial regional differences, deployment strategies should reflect local infrastructure, CCS availability, industrial priorities, and policy conditions rather than follow one universal model.

### Policy, governance, and long-term transition pathways

Figure 14 shows the increasing prominence of policy, economy, and decarbonization. Policy instruments should therefore reduce investment uncertainty, support shared infrastructure, create demand, and establish credible certification standards. However, incentives should be linked to full-chain greenhouse-gas emissions, capture effectiveness, methane leakage, storage permanence, and transparent reporting. The chronological evolution in Figs. 13 and 14 shows a transition from conventional SMR research toward CCS integration, renewable hybridization, sustainability assessment, and industrial deployment. Blue hydrogen should therefore be treated as a transitional pathway whose long-term role depends on improved capture performance, controlled methane emissions, viable infrastructure, and competitiveness with green hydrogen.

**Fig. 20:**
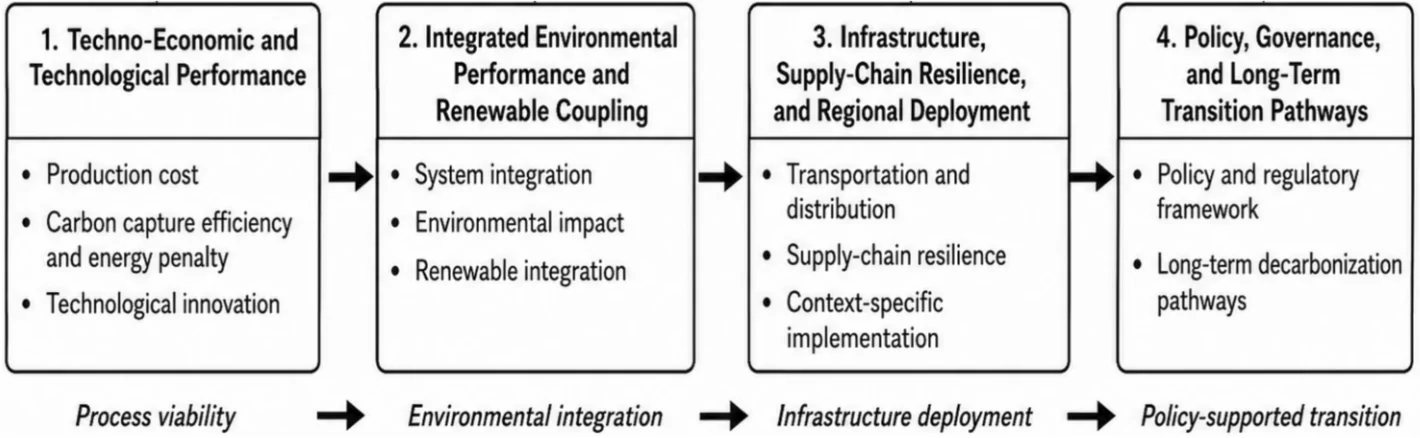
Integrated framework for the discussion, implications, and research outlook

Overall, the findings demonstrate that feasibility depends on the interaction of several technical, environmental, infrastructural, and institutional factors. To provide a clear and logically structured interpretation of these interdependencies, the discussion is organized into four interconnected dimensions: (1) techno-economic and technological performance, (2) integrated environmental performance and renewable coupling, (3) infrastructure, supply-chain resilience, and regional deployment, and (4) policy, governance, and long-term transition pathways. As illustrated in Fig. 20, these dimensions represent a sequential progression from process viability and environmental integration to infrastructure deployment and policy-supported transition. This integrated framework emphasizes that production cost, carbon-capture efficiency, environmental impacts, renewable-energy integration, transportation infrastructure, regional conditions, supply-chain resilience, and regulatory support must be evaluated collectively. Future studies should therefore combine process engineering, techno-economic analysis, life-cycle assessment, infrastructure planning, policy analysis, resilience modelling, and stakeholder decision-making to determine where, when, and under what conditions blue hydrogen can contribute effectively to industrial decarbonization and the broader transition toward low-carbon energy systems. Figure 20 summarizes the resulting progression from process viability and environmental integration to infrastructure deployment and policy-supported transition.

## Conclusions

The bibliometric analysis of 1432 research and review articles from the WOSCC (2000–2025) reveals growth in blue hydrogen research with an average annual growth rate of 17.6% since 2015. The People’s Republic of China leads global research output with 472 publications. It is followed by the United States with 190 publications, India with 133, and South Korea with 107. The Chinese Academy of Sciences is the most productive institution, with 50 publications and an h-index of 20. Centrality analysis identified CAS, CNRS, and the U.S. Department of Energy as influential institutions. The University of Calgary and the University of Regina also show growing contributions to CO₂-capture and hydrogen research. The most influential papers were authored by Ishaq et al. (2022), X.-Y. Yu et al. (2016), and Concepcion et al. (2009) while the International Journal of Hydrogen Energy is the leading publication source. CiteSpace and Bibliometrix analyses further revealed a transition in research focus from SMR toward CCS integration and renewable-driven hybrid hydrogen systems, with dominant themes including engineering, environmental science, materials, chemistry and sustainability-oriented hydrogen production research.

Thematic content analysis based on the keyword dendrogram and MCA identified seven major research themes, including hydrogen production technologies, CCS integration, renewable energy coupling, sustainability assessment, techno-economic analysis, catalyst development, and future energy transition pathways. The findings indicate that blue hydrogen research has evolved into a mature, interdisciplinary, and deployment-oriented research field driven by growing global interest in low-carbon energy transition and industrial decarbonization. The dominant research paradigm is centered on SMR or ATR with CCS integration, techno-economic optimization, life-cycle assessment, renewable energy hybridization, and large-scale industrial deployment. Future research should strengthen collaboration among process engineering, carbon capture science, environmental assessment, energy economics, policy development, infrastructure planning, and digital modelling. Such collaboration could improve system efficiency, reduce emissions and production costs, strengthen supply-chain resilience, and accelerate the commercialization of low-carbon hydrogen systems.

Although the WOSCC is recognized as one of the acceptable academic databases, it may not cover all relevant publications. Some studies indexed elsewhere could have been omitted. Potential inconsistencies may exist within the source data. Authors might appear under multiple name variations, use different initials, or publish under alternate affiliations. Such discrepancies can lead to inaccuracies when assessing the productivity of authors, institutions, or countries. Data cleaning procedures such as removing duplicates and reclassifying documents were implemented to minimize errors. Residual inconsistencies in the database may still influence the results. Future studies could address these limitations by integrating data from multiple databases to enhance coverage and reliability. Although this study identifies major research challenges through bibliometric and thematic analyses, it does not establish their relative importance. Future studies may employ expert-based multi-criteria decision-making approaches to prioritize these challenges and evaluate their relative importance.

## Supplementary Information

Below is the link to the electronic supplementary material.

Supplementary file 1.

## Data Availability

No datasets were generated or analysed during the current study.
