# Supplementary material for: A scientometric review of blue hydrogen production
Source: Discov Energy. 2026 Oct 17;6(1):87. doi: 10.1007/s43937-026-00203-x (PMC13676539; doi:10.1007/s43937-026-00203-x)
Supplement: Supplementary file 1 [file 43937_2026_203_MOESM1_ESM.docx]

### 2.3.1 VOSviewer (1.6.20)

VOSviewer is used to construct and visualize co-authorship, keyword co-occurrence, and institutional collaboration networks. Two researchers, Nees Jan Van Eck and Ludo Waltman, at Leiden University in the Netherlands, created VOSviewer a tool for scientometric and knowledge graph analysis [56]. This employs a mapping technique comparable to multidimensional scaling and represents the results clearly. Its objective function is mathematically represented in Equation (1) [57].

$F=\sum_{i=1}^{n} \sum_{j=1, j\neq i}^{n} S_{ij} . d_{ij}^{2}$	(1)

Here, F is the objective function value for the entire network, $S_{ij}$ is similarity between nodes $i$ and $j$ . Here $d_{ij}$is Euclidean distance between nodes $i$ and $j$ in two-dimensional space.

$d_{ij}= \sqrt{\left( x_{i}-x_{j} \right)^{2}+ \left( y_{i}-y_{j} \right)^{2}}$	(2)

VOSviewer offers a specialized approach for visualizing knowledge graph density maps. Equation 3 knowledge graph density maps show the measure.

$\frac{2}{n(n-1)} \sum_{i=1}^{n-1} \sum_{j=i+1}^{n} \sqrt{\left( x_{i1}-x_{j1} \right)^{2}+ \left( x_{i2}- x_{j2} \right)^{2}}=1$	(3)

Here n is the total number of nodes in the network. ($x_{i1}$, $x_{i2}$) and ($x_{i2}$, $x_{j2}$) are coordinates of nodes $i$ and $j$ in 2D space. $\sqrt{\left( x_{i1}-x_{j1} \right)^{2}+ \left( x_{i2}- x_{j2} \right)^{2}}$ is Euclidean distance $d_{ij}$ between nodes $i$ and $j$. the total of these distances is normalized by the factor $\frac{2}{n(n-1)}$ to maintain an average distance across all node pairs. VOSviewer offers a unique feature for visualizing knowledge graph density maps, setting it apart from other knowledge graph tools. The density map, denoted as $D\left( x \right)$ for a node located at coordinates $x \left( x_{1}, x_{2} \right)$ is computed using a weighted kernel function. This calculation considers the spatial distribution of all nodes in the graph, their associated weights, and a smoothing parameter.

$D\left( x \right)= \sum_{i=1}^{n} w_{i}. K. \left( \frac{\sqrt{\left( x_{i1}-x_{j1} \right)^{2}+ \left( x_{i2}- x_{j2} \right)^{2}}}{h. \phi} \right)$	(4)

The average pairwise distance $\phi$ represents the mean Euclidean distance between every unique pair of nodes in the network. It serves as a normalization factor, ensuring the density map accurately reflects relative node proximity regardless of the graph's overall scale [58,59].

$\phi= \frac{2}{n(n-1)} \sum_{i=1}^{n-1} \sum_{j=i+1}^{n} \sqrt{\left( x_{i1}-x_{j1} \right)^{2}+ \left( x_{i2}- x_{j2} \right)^{2}}$	(5)

Total Link Strength (TLS) is measured to calculate the intensity of connections between items in the bibliometric network, such as authors, organizations, or keywords. TLS represents the sum of the strengths of all links that a given item has with other items which quantifies both the extent and weight of its collaborations. Mathematically, TLS for item $i$ is expressed as:

${TLS}_{i}= \sum_{j=1}^{n} W_{ij}$	(6)

where $W_{ij}$ is the weight of the link between item $i$ and item $j$, and $n$ is the total number of items connected to $i$. Higher TLS value indicates stronger or more frequent connections which means the centrality and collaborative influence of that item within the research network. The occurrence percentage was calculated to determine the relative frequency of keywords within the dataset. This measure indicates how often a specific keyword appears compared to the total number of keyword occurrences, thereby reflecting its proportional importance in the research field. The occurrence $\%$ was computed using the following formula.

$Occurence \%= \frac{\mathrm{Occurrenc}e_{i}}{\sum_{k=1}^{n} \mathrm{Occur}_{k}} ⨯100$	(7)

where $Occurrence_{i}$ represents the number of times keyword $i$ appears, and $\sum_{k=1}^{n} {Occur}_{k}$ is the total number of keyword occurrences across the dataset. This metric provides a relative weight of keywords, which helps in identifying dominant themes and trends. The H-index was applied to evaluate the scientific impact and productivity of authors in the dataset. Following the WOSCC standard, all publications were ranked in descending order of citation counts. The H-index is then defined as the maximum value of *h* such that the author has at least *h* publications, each with at least *h* citations. Mathematically,

$h=\max\{ k\in\left\{ 1,2,3,\ldots\ldots n \right\} : c\left( k \right) \geq k\}$	(8)

Here $c\left( k \right)$ denotes the number of citations of the publication ranked at position $k$. This indicator captures both productivity (number of publications) and impact (citations), and is widely used to assess author-level influence in bibliometric studies. To normalize citation performance across publications of different ages, the citations per year (C/Y) metric was calculated. This indicator measures the average annual citation rate of an article, providing a fair comparison between older and newer publications. The formula applied is as follows:

$\frac{C}{Y}= \frac{Total Citation}{Reference Year-Publication year+1}$	(9)

Here, the total number of citations for an article as indexed in WOSCC was divided by the number of years since its publication with one year added to include the year of publication. This approach ensures that both recent and long-standing articles can be evaluated consistently in terms of their yearly citation impact. These themes are grouped into broader clusters. Concept mapping is applied to visually arrange the clusters and show the links among them (Novak & Cañas, 2008). Thematic mapping is used to guide the process of grouping related themes into broader domains that represent the knowledge structure ([61].

### 2.3.2 CiteSpace (6.1.R2 Advanced)

CiteSpace 6.1.R2 Advanced can construct and visualize knowledge networks and evaluate the structural quality of clusters using metrics such as modularity and silhouette values to identify the intellectual structure and evolution of a research field [62–64]. To examine the evolution of research domains within blue hydrogen production, a subject category timeline analysis was conducted. The WOSCC dataset was imported in CiteSpace and applied a time-slicing algorithm to capture longitudinal developments. Each node in the timeline represents a subject category, while the link between nodes denotes the co-occurrence or citation connection strength [65]. The software employs network pruning (pathfinder and pruning sliced networks) to reduce redundancy and enhance the clarity of knowledge clusters [66,64]. Modularity (Q) value is computed using Equation 10 to evaluate the clustering quality.

$Q= \sum_{i=1}^{k} \left( \frac{l_{i}}{L}-\left( \frac{d_{i}}{2L} \right)^{2} \right)$	(10)

Where, $l_{i}$ number of links between nodes within cluster $i$, $d_{i}$ is sum of degrees of all nodes in cluster $i$, total number of links in the entire network $L$.

### 2.3.3 Bibliometrix

Bibliometrix (R package) computes bibliometric indicators, build collaboration, co-word networks, and generate mapping visualizations like trends, dendrograms, conceptual maps for quantitative literature analysis [67–70]. This tool enables comprehensive quantitative analysis and visualization of scientific literature. To examine the thematic evolution of hydrogen research, bibliometric analysis was performed using the Bibliometrix package (version 4.2) in R. The dataset, retrieved from the WOSCC was analyzed to generate the centrality wheel of institutions, topic trend over the last 25 years, the keyword dendrogram, and the conceptual structure map based on Multiple Correspondence Analysis (MCA). Centrality measures how central or influential an institution is within the collaboration network [69]. The following equation is be used to measure Betweenness Centrality.

$C_{B}\left( i \right)=\sum_{s\neq i\neq t} \frac{\sigma_{st}(i)}{\sigma_{st}}$	(11)

Here $\sigma_{st}$ is the number of shortest paths between $s$ and $t$ and $\sigma_{st}(i)$ those passing through node $i$. This shows bridge institutions that connect research clusters. The Topic trend uses time-series frequency analysis of keyword occurrence [68].

$f_{t}= \frac{N_{t}}{\sum_{t=1}^{T} N_{t}}$	(12)

Here, $N_{t}$ is the number of keyword occurrence in year $t$ and $T$ is the total number of years. The dendrogram is built from a similarity or distance matrix between keywords, typically using Euclidean distance and Ward’s linkage method [70].

$d\left( x_{i}, x_{j} \right)= \sqrt{\sum_{k=1}^{p} {(x_{ik}-x_{jk})}^{2}}$	(13)

Here, $d\left( x_{i}, x_{j} \right)$ is the Euclidean distance between keyword $i$ and keyword $j$., $x_{ik}$ is the value (or frequency) of keyword $i$ on variable $k$. $x_{jk}$ is the is the value (or frequency) of keyword $j$ on variable $k$. $p$ is the number of dimensions or features, typically the number of other keywords or documents considered. This equation measures how different or similar two keywords are based on how often they appear together. Ward’s linkage minimizes within-cluster variance as mentioned in equation 14.

$D\left( A,B \right)= \frac{\left| A \right||B|}{\left| A \right|+|B|}\left| \left| x_{A}- x_{B} \right| \right|^{2}$	(14)

Here $A, B$ are the two clusters being merged during hierarchical clustering. $\left| A \right|$, $|B|$ are the number of elements (keywords) in each cluster. $x_{A}$, $x_{B}$ are the squared Euclidean distance between cluster centroids. Multiple Correspondence Analysis is a multivariate statistical method that projects categorical data (keywords) into a low-dimensional conceptual space [67]. The following equation is used to measure MCA. This is used to identify thematic grouping.

$I\left( f \right)= \sum_{i,j} \frac{{(f_{ij}-f_{i}. f_{j})}^{2}}{f_{i}.f_{j}}$	(15)

Here, $f_{ij}$is the is the joint frequency of categories $i$ and $j$ and $f_{i}$ and $f_{j}$ are marginal frequencies.

**References**

[56]	Van Eck NJ, Waltman L. Software survey: VOSviewer, a computer program for bibliometric mapping. Scientometrics 2010;84:523–38. https://doi.org/10.1007/s11192-009-0146-3.

[57]	Van Eck NJ, Waltman L. VOS: A New Method for Visualizing Similarities Between Objects. In: Decker R, Lenz H-J, editors. Advances in Data Analysis, Berlin, Heidelberg: Springer Berlin Heidelberg; 2007, p. 299–306. https://doi.org/10.1007/978-3-540-70981-7_34.

[58]	Wang H, Liu H, Yao J, Ye D, Lang Z, Glowacz A. Mapping the knowledge domains of new energy vehicle safety: Informetrics analysis-based studies. Journal of Energy Storage 2021;35:102275. https://doi.org/10.1016/j.est.2021.102275.

[59]	Van Eck NJ, Waltman L, Noyons ECM, Buter RK. Automatic term identification for bibliometric mapping. Scientometrics 2010;82:581–96. https://doi.org/10.1007/s11192-010-0173-0.

[60]	Novak JD, Cañas AJ. The theory underlying concept maps and how to construct and use them 2008.

[61]	Braun V, Clarke V. Using thematic analysis in psychology. Qualitative Research in Psychology 2006;3:77–101. https://doi.org/10.1191/1478088706qp063oa.

[62]	Du Q, Zhao R, Wan Q, Li S, Li H, Wang D, et al. Protocol for conducting bibliometric analysis in biomedicine and related research using CiteSpace and VOSviewer software. STAR Protocols 2024;5:103269. https://doi.org/10.1016/j.xpro.2024.103269.

[63]	Xu G, Yang Q, Li Q, Yu H. Research of digital management on sport: An analysis of bibliometrics using CiteSpace Software. Medicine 2024;103:e40872. https://doi.org/10.1097/MD.0000000000040872.

[64]	Chen C. CiteSpace II: Detecting and visualizing emerging trends and transient patterns in scientific literature. J Am Soc Inf Sci 2006;57:359–77. https://doi.org/10.1002/asi.20317.

[65]	Zhao X, Nan D, Chen C, Zhang S, Che S, Kim JH. Bibliometric study on environmental, social, and governance research using CiteSpace. Front Environ Sci 2023;10:1087493. https://doi.org/10.3389/fenvs.2022.1087493.

[66]	Zhao Y, Liu Q, Duan Y, Zhang Y, Cui Y, Huang Y, et al. Hydrogen energy deployment in decarbonizing transportation sector using multi-supply-demand integrated scenario analysis with nonlinear programming — A Shanxi case study. International Journal of Hydrogen Energy 2022;47:19338–52. https://doi.org/10.1016/j.ijhydene.2022.04.102.

[67]	Ismail A, Munsi H, Yusuf AM, Hijjang P. Mapping One Decade of Identity Studies: A Comprehensive Bibliometric Analysis of Global Trends and Scholarly Impact. Social Sciences 2025;14:92. https://doi.org/10.3390/socsci14020092.

[68]	Chen X, Xie H, Tao X, Xu L, Wang J, Dai H, et al. A topic modeling‐based bibliometric exploration of automatic summarization research. WIREs Data Min & Knowl 2024;14:e1540. https://doi.org/10.1002/widm.1540.

[69]	Duggan EW, Atwood GS, Sanford JA, Tsai MH, Egbaria JK, Carmichael-Tanaka N, et al. Using Bibliometric Data to Define and Understand Publishing Network Equity in Anesthesiology. Anesthesia & Analgesia 2024;139:944–54. https://doi.org/10.1213/ANE.0000000000006877.

[70]	Wei W, Jiang Z. A bibliometrix-based visualization analysis of international studies on conversations of people with aphasia: Present and prospects. Heliyon 2023;9:e16839. https://doi.org/10.1016/j.heliyon.2023.e16839.
